# Poly(A) Binding Protein 1 Enhances Cap-Independent Translation Initiation of Neurovirulence Factor from Avian Herpesvirus

**DOI:** 10.1371/journal.pone.0114466

**Published:** 2014-12-11

**Authors:** Abdessamad Tahiri-Alaoui, Yuguang Zhao, Yashar Sadigh, James Popplestone, Lydia Kgosana, Lorraine P. Smith, Venugopal Nair

**Affiliations:** 1 The Pirbright Institute, Ash Road, Pirbright, Woking, Surrey, United Kingdom; 2 The Division of Structural Biology, The Wellcome Trust Centre for Human Genetics, Oxford University, Oxford, United Kingdom; University of British Columbia, Canada

## Abstract

Poly(A) binding protein 1 (PABP1) plays a central role in mRNA translation and stability and is a target by many viruses in diverse manners. We report a novel viral translational control strategy involving the recruitment of PABP1 to the 5' leader internal ribosome entry site (5L IRES) of an immediate-early (IE) bicistronic mRNA that encodes the neurovirulence protein (pp14) from the avian herpesvirus Marek’s disease virus serotype 1 (MDV1). We provide evidence for the interaction between an internal poly(A) sequence within the 5L IRES and PABP1 which may occur concomitantly with the recruitment of PABP1 to the poly(A) tail. RNA interference and reverse genetic mutagenesis results show that a subset of virally encoded-microRNAs (miRNAs) targets the inhibitor of PABP1, known as paip2, and therefore plays an indirect role in PABP1 recruitment strategy by increasing the available pool of active PABP1. We propose a model that may offer a mechanistic explanation for the cap-independent enhancement of the activity of the 5L IRES by recruitment of a *bona fide* initiation protein to the 5' end of the message and that is, from the affinity binding data, still compatible with the formation of ‘closed loop’ structure of mRNA.

## Introduction

All known viruses share an absolute requirement for host cell ribosomes and are exquisitely dependent on cellular translation factors to meet their synthetic needs. Faced with this dependency, viruses have evolved strategies to commandeer the host translational apparatus [1], [2]. Studies of viral subversion of host protein synthesis machinery have not only revealed key steps in viral pathogenesis, but also defined paradigms for translational control in uninfected cells [2].

Poly(A) binding protein 1 (PABP1), also known as cytoplasmic PABPC1, is a central regulator of gene expression by virtue of its multiple roles in mRNA translation and stability [3]. In coordination with other initiation factors such as eIF4G, PABP1 is known to bridge both ends of mRNA to form a ‘closed loop’ topology [4] which may promote translation initiation by enhancing ribosome recruitment [5]. The high abundance of PABP1 in the cytosol, its highly conserved nature and its central role in global protein translation make it a common target by many viruses in diverse manners [6]. For example, PABP1 is cleaved by virally encoded proteases from members of the single stranded RNA *Picornaviridae* family as a mechanism of host protein synthesis shutoff [7], [8]. Alternatively, some reoviruses encode proteins that inhibit PABP1-eIF4F interaction leading to host protein synthesis shutoff and nuclear localization of PABP1 [9], [10]. Bunyaviruses [11] and some herpesviruses such as HSV-1 (herpes simplex virus type 1) and KSHV (Kaposi’s sarcoma-associated herpesvirus) have also been reported to redistribute PABP1 to the nucleus [12]–[14]. The mechanisms behind relocalisation of PABP1 to the nucleus are still an open debate [15], [16]. By contrast, PABP1 does not accumulate in the nucleus of cells infected with the herpesvirus HCMV (human cytomegalovirus), but instead is recruited to eIF4F complex [17], [18]. Recently, it was shown that PABP1 is induced by the HCMV gene product, UL38, a target of rapamycin complex 1 (mTORC1) activator [19].

In this paper we report a novel transcript-specific translation control strategy involving the recruitment of PABP1 to an internal poly(A) sequence within the 5' leader (5L) internal ribosome entry site (IRES) of an immediate-early (IE) transcript from the avian herpesvirus Marek’s disease (MD) virus serotype 1 (MDV1). The IE transcript encodes the phosphorylated protein pp14, a viral protein that we have recently identified as a neurovirulence factor from MDV1 [20]. MD is a major illness of poultry worldwide that causes disseminated visceral T cell lymphomas and neurological manifestations in infected chicken [21]. Our finding provides mechanistic explanation of how a key viral transcript is translated efficiently by using an enhancer internal poly(A) sequence within the 5L IRES, and exploits the intrinsic property of PABP1 as a *bona fide* initiation factor. Additionally, using a combination of RNA interference analysis and reverse genetic mutagenesis, we demonstrate that a subset of virally-encoded microRNAs (miRNAs) target the inhibitor of PABP1, known as paip2, thus increasing the availability of an active pool of PABP1 and indirectly enabling PABP1 recruitment strategy. We propose a model that may offer mechanistic explanation for the cap-independent enhancement of the activity of the 5L IRES by recruitment of a *bona fide* initiation factor to the 5' end of the message and that is, from affinity binding data, still compatible with the formation of ‘closed loop’ structure of mRNA.

## Results

### Internal poly(A) within the 5L IRES of MDV1-pp14 mediates PABP1 recruitment and IRES function

**Figure 1:**
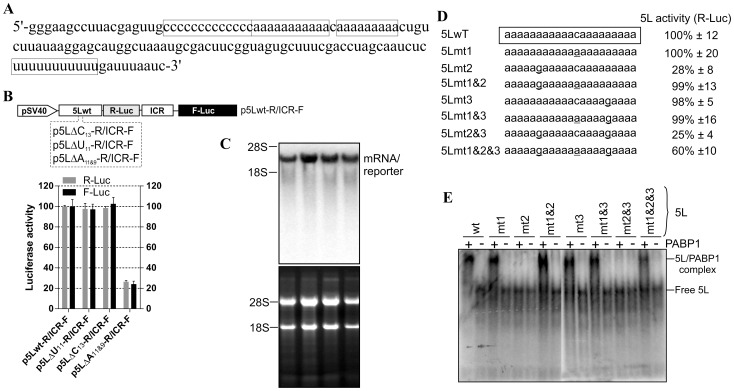
Effect of deletions of the internal homo-polymeric sequences on the activity of the 5L IRES and mutation analysis of affinity interaction between the 5L IRES and PABP1. (A) Partial sequence from the full length 5L IRES from the immediate-early 1.8-kb mRNA that encodes pp14b isoform from Marek’s disease virus serotype 1. The 5L IRES spans nucleotides 129339-129798 (acc: AF243348). The internal poly-pyrimidine sequences C_13_ and U_11_ are boxed as well as the internal poly(A) sequences A_11_ and A_9_. (B) Schematic of the DNA constructs used for the luciferase reporter assay. In this vector the translation of R-Luc is controlled by the 5L IRES whereas the F-Luc is under the control of the intercistronic IRES (ICR). This configuration mimics the dual IRES bicistronic 1.8-kb mRNA from MDV1. DF-1 cells were transiently transfected with the indicated DNA vectors and after 24 h the cells were lysed and the luciferase activities were measured. The results are expressed as per cent change in luciferase activity relative to the control wild type sequence (5Lwt). (C) Northern blotting was performed on total RNA extracted from cells transfected with DNA constructs depicted in B. Hybridization was done with a random-primed 32P-labelled DNA fragment corresponding to the 5' end of the F-Luc open reading frame. Ethidium bromide-staining of the gel used for Northern blot is shown below the blot with 18S/28S rRNAs as size markers and loading control. (D) The mutated nucleotides within the internal poly(A) from the 5L IRES are underlined. The corresponding DNA vectors were used to transfect DF-1 cells as described in B. For simplicity, only the R-Luc values are shown as the F-Luc follows the same trend due to the coevolved synergistic functional relationship between the 5L IRES and the ICR IRES. The results are expressed as per cent change relative to the control wild type sequence (5Lwt). The experiment was repeated three times and the SEM is shown. (E) Purified recombinant human PABP1 (0.5 µM) was incubated with ^32^P-end labelled 5L IRES RNAs from wild type or from the indicated mutants and separated on a native 6% polyacrylamide gel by Electrophoretic Mobility Shift Assay (EMSA). It should be noted that the RNA was obtained by in vitro transcription and that it has no 3' poly (A) tail. The complex between PABP1 and the 5L IRES RNA was visualized by autoradiography using phosphor screen. The complex 5L IRES/PABP1 is observed in all combinations except with the mutants 5Lmt2 and 5Lmt2&3.

We have previously reported the presence of a functional IRES within the 5' leader of an IE mRNA from MDV1 [22]. The 5L IRES is part of a naturally occurring bicistronic mRNA that contains another functional IRES within the inter-cistronic region [23]. We showed that both IRES elements function synergistically and proposed an allosteric model for their activity [22]. The 5L IRES controls the expression of viral pp14 that we have recently shown to mediate the neurovirulence phenotype of MDV1 [20]. An important feature of the sequence of the 5L IRES is that it contains two sets of internal poly-pyrimidine sequences; one is C_13_ and the second is U_11_ (Fig. 1A). In addition there are two adjacent poly(A) sequences, A_11_ and A_9_ that are separated by one cytosine (Fig. 1A). Deletion of the C_13_ and of the U_11_ does not affect the activity of the 5L reporter, however, deletion of the A_11_ and A_9_ poly(A) reduces the activity of the reporter by more than 75% (Fig. 1B), and these effects are unlikely to be due to altered RNA stability or abundance as indicated from Northern blot analysis (Fig. 1C). The concomitant reduction of the ICR IRES activity does not indicate nonspecific effect but is a manifestation of the coevolved functional relationship between the two IRESes that we have previously reported [22]. To further investigate the role of each of the internal poly(A) sequences in the activity of the 5L IRES within the reporter mRNA, we made single mutations as depicted in (Fig. 1D). The constructs were designed in a configuration that mimics the naturally occurring viral bicistronic dual IRES [22] and were used to transfect DF-1 cells. After 24 hours incubation the luciferase activities were measured (Fig. 1D). For simplicity only the activity of the R-Luc that is under the control of the 5L IRES is shown; as the activity of the F-Luc (controlled by the ICR IRES) followed the same trend. Mutating the internal C to A in the 5Lmt1 did not affect the activity of the 5L reporter that remained similar to the control 5Lwt. In the 5Lmt2, where the A_11_ tracts were disturbed by mutating the middle A to G, the activity of the 5L reporter decreased by about 80%. Combining mutations mt1 and mt2 within the construct 5Lmt1&2 restored the activity of the 5L reporter to its wild type level; suggesting that A_11_ is the optimal requirement for maintaining full 5L IRES activity within reporter mRNA. This is supported by the results from the constructs 5Lmt3, 5Lmt1&3, 5Lmt2&3 and 5Lmt1&2&3 (Fig. 1D).

**Figure 2:**
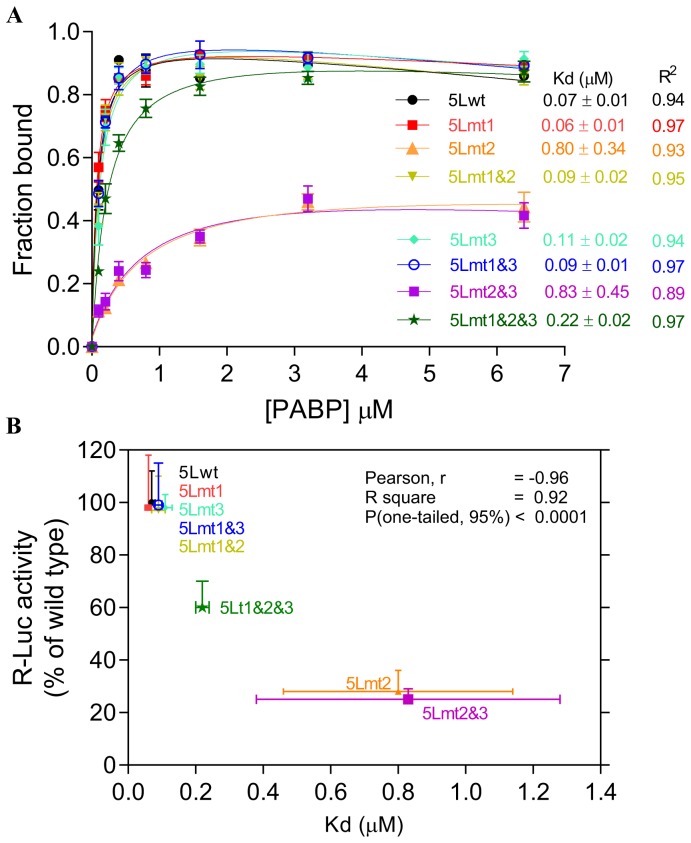
Effect of mutations within the internal poly(A) sequences on the binding affinities of the 5L IRES to the PABP1. (**A**) Binding affinities between recombinant PABP1 and ^32^P-end labelled wild type and mutant 5L IRES sequences showing the fraction bound for each concentration of PABP1. The apparent dissociation constants are shown to the right with SEM from three repeats. (**B**) Correlations between binding affinities in panel ***A*** and the R-Luc activity as determined by transfection and reporter assay from panel ***D*** of Fig. 1.

**Figure 3:**
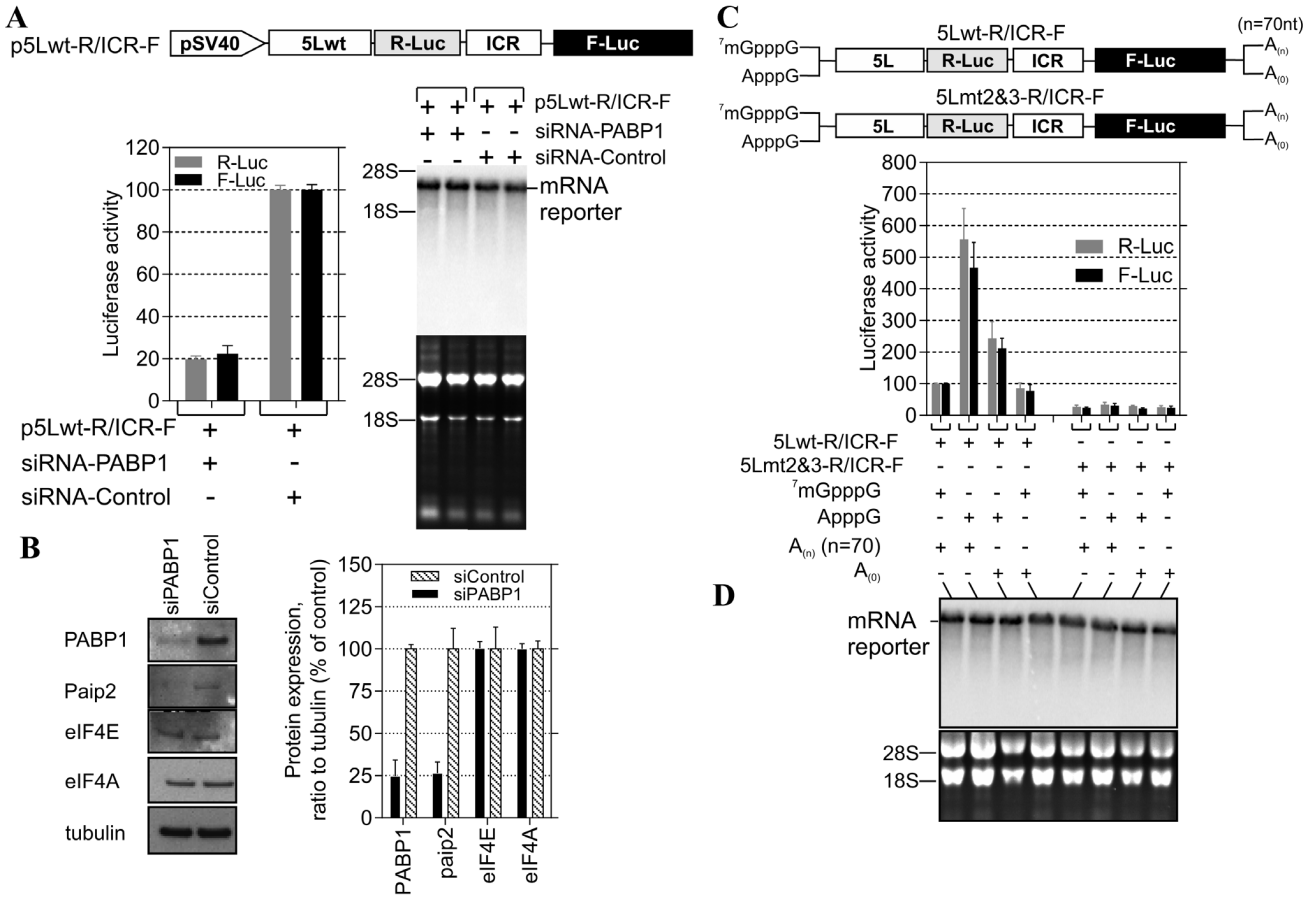
PABP1 knockdown and functional analysis of the interplay between 5L IRES internal poly(A) and poly(A) tail. (**A**) DF-1 cells were co-transfected with the depicted DNA constructs and with the PABP1 siRNA or nonsilencing siRNA control. The cells were lysed after 48 h incubation and used for luciferase assays. The results are presented as per cent change relative to nonsilencing siRNA control. The experiment was performed six times and the error bars indicate the SEM. Northern blotting was performed on total RNA extracted from transfected cells. Hybridization was done with a random-primed 32P-labelled DNA fragment corresponding to the 5' end of the F-Luc open reading frame. Ethidium bromide-staining of the gel used for Northern blot is shown below the blot with 18S/28S rRNAs as size markers and loading control. (**B**) Total proteins were harvested 48 h posttransfection and analysed by immunoblotting with the indicated antibodies. Quantification of the immunoblots from panel ***B*** using ImageQuant software is shown to the right. (**C**) DF-1 cells were transfected for 1 h with the indicated bicistronic dual IRES mRNA reporters and subsequently washed (0 hour); then 6 hours posttransfection the luciferase activity was measured and expressed as per cent change relative to capped and polyadenylated 5Lwt-R/ICR-F mRNA. The experiment was performed four times and the error bars indicate the SEM. (**D**) Total RNA was extracted from the transfected cells and the integrity of the bicistronic dual IRES mRNA reporters was analysed by Northern blotting and ^32^P-lablled probe against R-Luc, followed by phosphor screen autoradiography. As in panel ***A***, Ethidium bromide-staining of the gel used for Northern blot is shown below the blot with 18S/28S rRNAs as size markers and loading control.

The internal poly(A) has the potential to interact with the PABP1 and therefore modulates the activity of the 5L IRES. To show that the internal poly(A) has indeed the ability to interact with PABP1 we performed electrophoretic mobility shift assay (EMSA) and demonstrated the occurrence of 5Lwt IRES/PABP1 complex that is seen in all combinations except with 5Lmt2&3 and 5Lmt2 that display poor 5L reporter activity (Fig. 1D and 1E). Our mutation analyses and EMSA are in agreement with previous findings [24] that showed A_11_ and A_12_ are capable of competing effectively with A_25_ for PABP1, whereas A_9_ and A_10_ are not. To further demonstrate the specificity of the interaction between internal poly(A) and PABP1, we performed affinity binding assays (Fig. 2A). The recombinant PABP1 binds tightly to the 5Lwt and to all mutants except when the length the internal poly(A) is reduced to less than 10-nucleotides, see for example 5Lmt2 and 5Lmt2&3 (Fig. 2A), and the binding affinity does not significantly change when the internal poly(A) length increases beyond A_11_, compare for example 5Lwt and 5Lmt1 (Fig. 2A). There is a good correlation between the binding affinity of PABP1 to the internal poly(A) and the activity of the 5L reporter (Fig. 2B). The importance of PABP1 for the activity of the 5L reporter was further demonstrated by siRNA-mediated PABP1 depletion. DF-1 cells were co-transfected with siRNA that targets PABP1 or control siRNA and with the reporter construct depicted in (Fig. 3A). Following 48 hour incubation the activity of the 5L reporter was assessed by measuring the luciferase activities from R-Luc and F-Luc. There is 80% decrease in the activity of the 5L reporter in the siPABP1 as compared to the control siRNA (Fig. 3A). When we used a reporter construct that lacks the 5L IRES and in which the R-Luc is under canonical cap-dependent translation and the F-Luc under ICR IRES control (S1A Figure, pR/ICR-F reporter), we found that siRNA-mediated PABP1 depletion caused only about 40% reduction in R-Luc activity with no apparent effect on the activity of F-Luc which is now controlled by the ICR IRES (S1A Figure), indicating the specific effect of PABP1 depletion on the activity of the 5L reporter. Northern blotting analysis shows that the decrease in the activity of the 5L reporter is not due to the stability or abundance of the reporter mRNAs (Fig. 3A and S1A Figure). Immunoblotting analysis reveals ∼75% decrease in the level of PABP1 in cells transfected with siPABP1, and as reported by another study [25] we also observed a concomitant decrease in the level of paip2, whereas the level of other translation factors such as eIF4E and eIF4A appeared unchanged (Fig. 3B). The above data indicate that PABP1 is involved in the regulation of the activity of the 5L IRES within the mRNA reporter, most likely via its interaction with the internal 5' poly(A). To gain further insights on how the PABP1 may mediate the regulation of the 5L IRES we investigated the interplay between the 3' end poly(A) tail, the internal poly(A) and the 5' cap structure using *in vitro* engineered mRNA reporters depicted in (Fig. 3C). The rationale behind this is that the activity of some cellular IRESes has been shown to be enhanced by poly(A) tail in the absence of PABP1 [26], [27]. Furthermore, we have previously shown that the 5L IRES efficiently initiates translation when cap-dependent translation initiation is inhibited [22]. Using RNA transfection experiments we show here that when the cap structure (^7^mGpppG) is replaced by the cap analogue (ApppG) the activity of the 5Lwt reporter increases by at least 5-fold in the presence of the poly(A) tail and only by ∼2-fold in the absence of the poly(A) tail (Fig. 3C). However, in the presence of the cap structure the absence of the poly(A) tail does not significantly alter the activity of the 5Lwt reporter (Fig. 3C), suggesting the possibility that the internal poly(A) within the 5L IRES may assume some of the functions of the poly(A) tail such as the direct recruitment of PABP1 to the 5L IRES located at the 5' end of the bicistronic mRNA reporter. Mutations within the internal poly(A), for example 5Lmt2&3, that simultaneously reduce the activity of the 5L reporter and the binding of the PABP1 also make the activity of the 5L reporter insensitive to the nature of the 5' and 3' ends of the bicistronic mRNA (Fig. 3C). In all the combinations tested the activity of the 5Lmt2&3 reporter is reduced by more than 70% (Fig. 3C, 5Lmt2&3-R/ICR-F). The importance of the interplay between the 3' poly(A) tail, the internal poly(A) within the 5L IRES for the activity of the 5L reporter was further demonstrated by using reporter mRNA that lacks the 5L IRES and in which the R-Luc is under canonical cap-dependent translation and the F-Luc under ICR IRES control (S1B Figure, R/ICR-F mRNA reporter). The data show that the activity of the ICR IRES is slightly enhanced in the absence of both the cap-structure and the 3' poly(A) tail and as expected the R-Luc activity which is now under canonical cap-dependent translation initiation was severely impaired in the absence the cap structure and the 3' poly(A) tail (S1B Figure). Northern blotting shows that variations in the activity of the 5L reporter are not due to the stability or the abundance of the reporter mRNAs (Fig. 3D and S1B Figure). These results indicate that the internal poly(A) and the poly(A) tail may work in synergy to enhance the activity of the 5L IRES within the mRNA reporter possibly by bridging the ends of the message in a cap-independent manner.

### MDV1 infection reduces paip2 expression but does not affect PABP1 localization or accumulation

**Figure 4:**
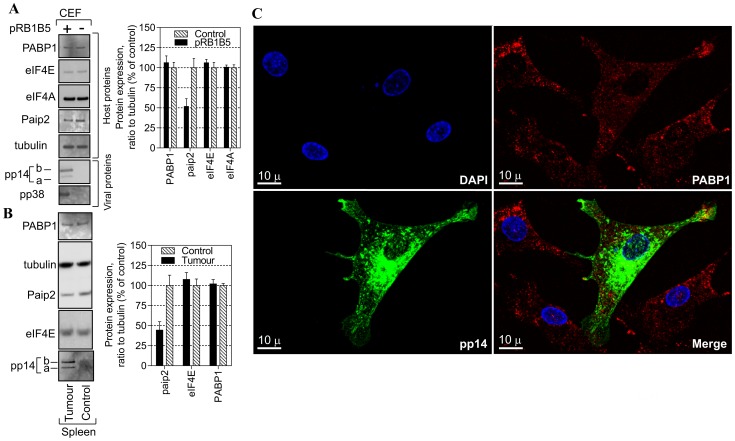
Effect of MDV1 infection on paip2 expression, PAPB1 level and localization. (**A**) Chicken embryo fibroblasts (CEF) were transfected with oncogenic BAC clone pRB1B5 of MDV1 or mock-transfected for 72 h. Total proteins were harvested and analysed by immunoblotting with the indicated antibodies. Quantification of the immunoblots from panel ***A*** using ImageQuant software is shown to the right. The results are from two independent experiments each in duplicate. (**B**) Total proteins were extracted from control samples or from samples taken from chicken infected with the oncogenic BAC clone pRB1B5 derived from archive samples. Proteins were analysed by immunoblotting as in panel ***A***. Quantification of the immunoblots from panel ***B*** using ImageQuant software is shown to the right. The results are repeats from two different archive samples derived from the same chicken challenge experiment. (**C**) Indirect immunofluorescence of pRB1B5-infected CEF 72 h posttransfection. A series of optical sections were taken sequentially for each channel along the z-axis using a step size of 0.290 µm. The resulting 3D confocal image was reconstructed using IMARIS software. DAPI-staining shows the nucleus in blue, PABP1 in red and pp14 in green, the scale bar: 10 µm.

Infection with some herpesviruses such as HSV1 [13], [14] and KSHV [12] can trigger PABP1 to accumulate in the nucleus. By contrast, in cells infected with HCMV, another related herpesvirus, PABP1 does not redistribute to the nucleus but accumulates in the cytoplasm and its level increases in HCMV-infected cells [17], [19]. Given the functional importance of PABP1 for the IRES-driven expression of the IE pp14, we investigated the effect of MDV1 infection on PABP1 expression. Total proteins were isolated from control and MDV1-infected samples and the overall abundance of selected viral and host proteins were evaluated by immunoblotting (Fig. 4A and S2A and S2B Figure). There is no apparent effect on the level of PABP1 expression in primary CEF 72 hour post-transfection with the BAC clone of the oncogenic pRB1B5 as compared to control cells (Fig. 4A). MDV1 infection does not appear to affect the level of other translation initiation factors such eIF4E and eIF4A despite successful viral replication and viral antigens expression (Fig. 4A). Similar results are seen in tumour vs. control tissues from chicken inoculated with RB1B5 strain of MDV1 (Fig. 4B). Interestingly, the level of PABP-interacting protein 2 (paip2) in both pRB1B5 CEF-transfected and pRB1B5-derived tumours is about 50% less than that detected in control samples (Fig. 4A-4B). Significantly, paip2 is well known to preferentially inhibit translation of poly(A)-containing mRNA by interdicting PABP1 function [28].

To examine the effect of MDV1 infection on PABP1 localization we used laser scanning confocal microscopy and indirect immunofluorescence (Fig. 4C). The intensity and distribution of PABP1 staining show similar patterns between MDV1-infected and adjacent non-infected control cells. The majority of PABP1 localizes to the cytoplasm (Fig. 4C). Examination of other translation initiation factors such as eIF4E, eIF4A and eIF4G shows that MDV1 infection does not affect their accumulation or cellular distribution (S3 Figure). Thus, MDV1 infection, like HCMV infection appears not to interfere with the global cytoplasmic localization of PABP1 but unlike HCMV, MDV1 does not appear to force the infected cells to increase the supply of PABP1 but may have evolved an alternative strategy that reduces the level of the paip2; the inhibitor of PABP1.

### Viral miRNAs repress paip2, the inhibitor of PABP1 to allow for optimal 5L IRES activity

**Figure 5:**
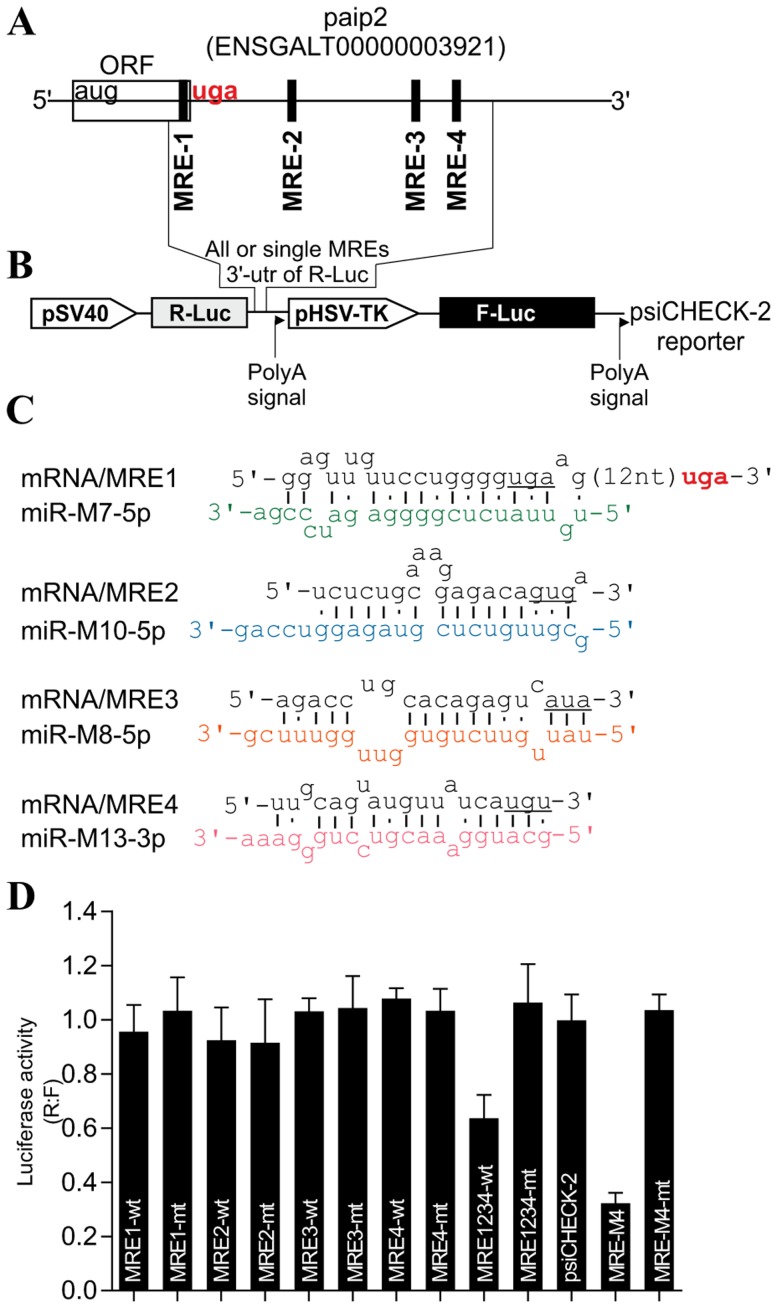
MDV1-encoded miRNAs target PABP1-interacting protein 2, paip2. (**A**) The paip2 transcript showing paip2 open reading frame (ORF), the 3' untranslated region (UTR) and the miRNA response elements (MRE). (**B**) Schematic of the reporter construct containing individual or combined MREs sequences downstream of the simian virus 40 promoter-driven *Renilla* luciferase cassette from psiCHECK-2 vector. (**C**) The predicted duplexes between paip2 mRNA and MDV1 miRNAs. The mutated nucleotides within the seed regions of paip2 mRNA are underlined. (**D**) Luciferase-based miRNA reporter assay. The full length region from the paip2 mRNA that contains all the MREs or the individual MREs and their mutated versions were made as synthetic oligonucleotides and sub-cloned into the sensor plasmid downstream of the *Renilla* luciferase in psiCHECK-2 vector. The resulting constructs were used to transfect MSB1; an MDV1-transformed CD4+ T-cell line derived from a spleen lymphoma induced by BC-1 strain of MDV1 constitutively expressing viral miRNAs. As positive control for assay validation we have used MRE-M4 that was previously shown to be targeted by MDV1 miRNA-M4. The normalized *Renilla* luciferase activities from five experiments are shown with the error bars (SEM) relative to that seen for the empty vector psiCHECK-2 which value is set to 1.

Next, we investigated the strategy by which MDV1 might mediate the decrease of paip2 protein accumulation. Using pattern-based algorithm for the discovery of miRNA target sites and the corresponding heteroduplexes [29], we identified several non-canonical sites within chicken paip2 mRNA as potential targets (Fig. 5A and 5C) for some of the previously published virally-encoded MDV1 miRNAs [30]–[32]. To assess the possible repressive effect of MDV1 miRNAs on paip2 we used sensor vector (Fig. 5B) in which the predicted microRNA response elements (MREs) or their mutated versions were cloned; either as individual MREs or as full length 3’UTR in the psiCHECK-2 vector [29]. The nucleotide sequences of individual MREs that were cloned in the sensor vector are shown in the supplementary data (S4 Figure). The resulting constructs were used to transfect MSB1; an MDV1-transformed CD4+ T-cell line derived from a spleen lymphoma induced by BC-1 strain of MDV1 [33] and that constitutively expresses MDV1 miRNAs [31]. As positive control for assay validation we used a MRE (MRE-M4) that was previously shown to be targeted by MDV1-miRNA-M4 which is an ortholog of the human miR155 [34]. Dual-Luciferase assays show significant repression in MSB1 transfected with the sensor vector carrying wild type MREs only when all of them are present within the native full length 3' UTR (MRE1234-wt) but not with the individual MREs (Fig. 5D). Examination of viral miRNA expression levels by TaqMan assay shows clear differences in their accumulation; with miR-M10 being the most highly expressed (S5 Figure). The high level of MDV1 miR-M10 expression does not necessarily correlate with its ability to mediate repression of the reporter gene on its own; suggesting that miRNA-mediated repression may require the synergistic action of all four virally-encoded miRNAs.

**Figure 6:**
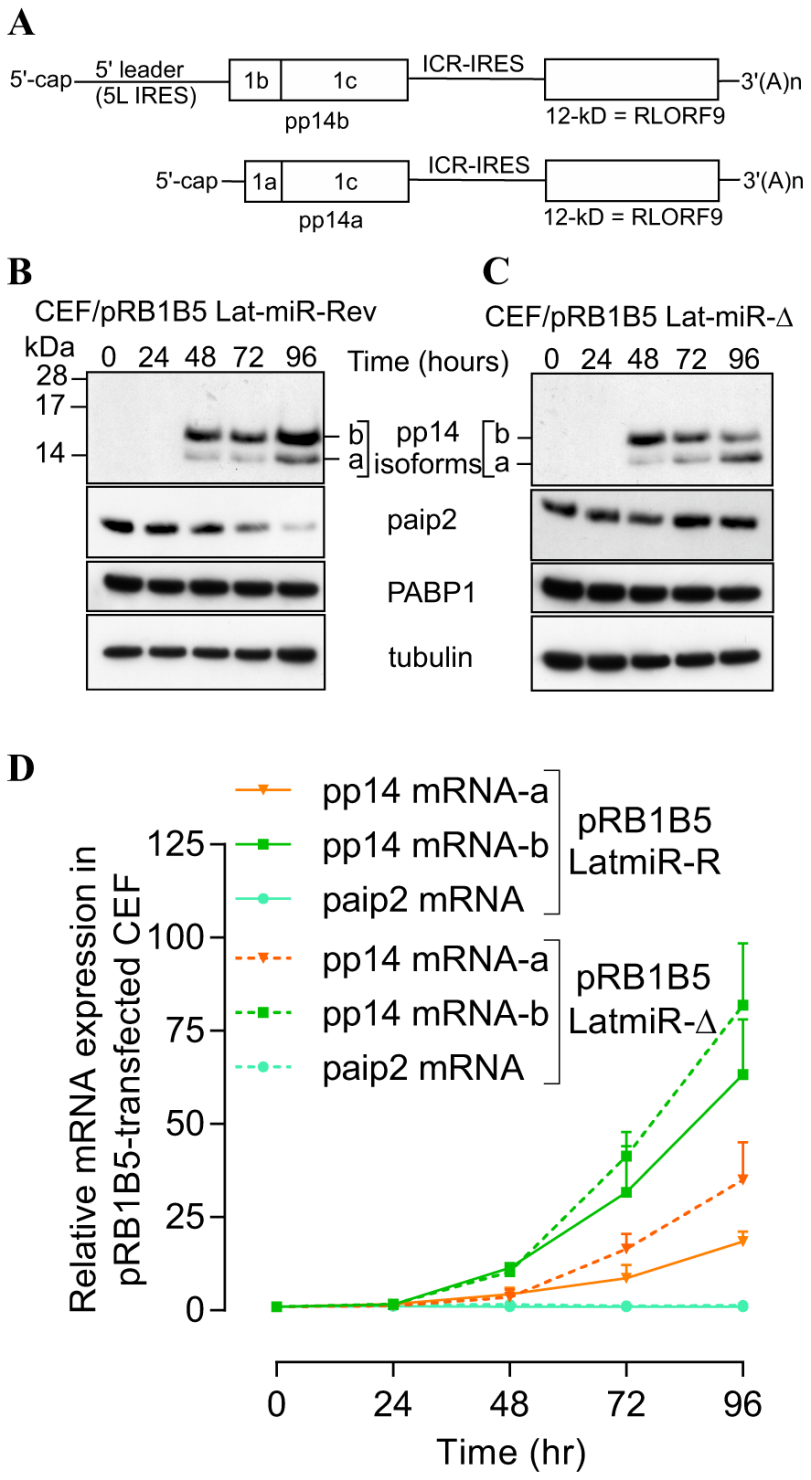
Reverse genetic mutation analysis shows that MDV1 miRNAs from Lat-cluster are responsible for paip2 repression. (**A**) Schematic representation of the bicistronic transcripts that we and others have cloned as cDNA and that encode for pp14a and pp14b isoforms, modified from Tahiri-Alaoui et al, J. Virol. Dec. 2009, Vol.83, No. 24, p12769-12778. (**B**) & (**C**) Chicken embryo fibroblasts (CEF) were transfected with BAC clone pRB1B5 Lat-miR-Revertant or pRB1B5 Lat-miR- deletion, respectively. RNA and proteins were simultaneously extracted using Trizol at the indicated time points. Viral and host proteins were detected by immunoblotting with the indicated antibodies. (**D**) Quantitative RT-PCR of host (paip2) and of viral transcripts (pp14a and pp14b isoforms) at the indicated time points. GAPDH is used as the endogenous control and time zero is used as the calibrator. All experiments were repeated three times and the error bars indicate the SEM.

**Figure 7:**
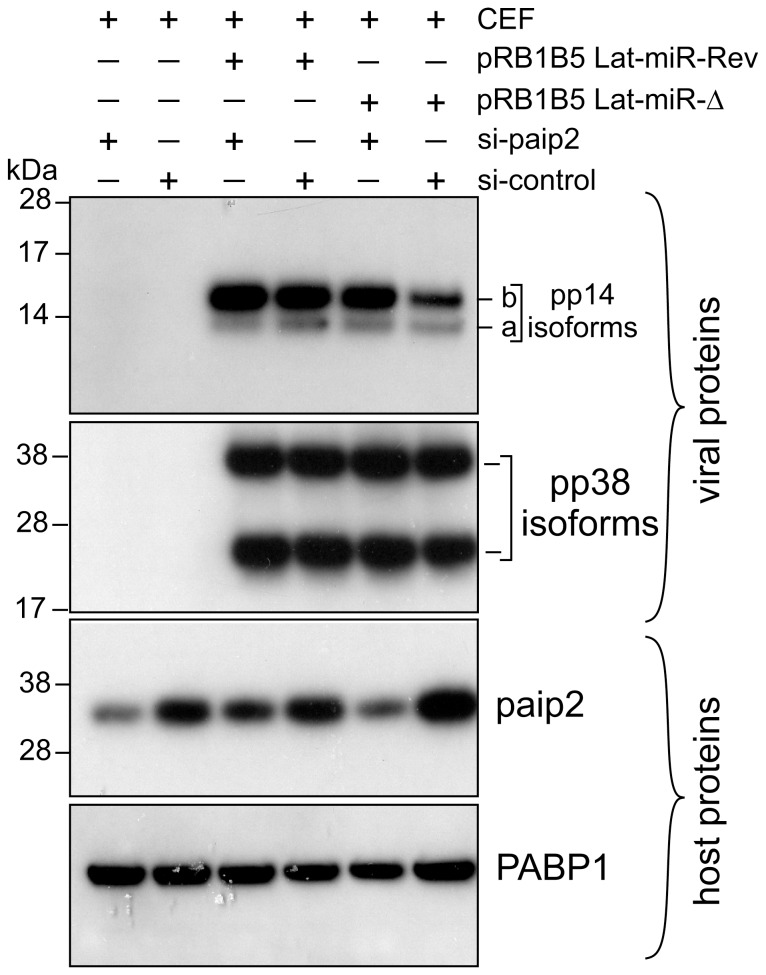
Depletion of paip2 by RNA interference in cells infected with Lat-miR BAC mutants. Chicken Embryo Fibroblasts were co-transfected with BAC clone of pRB1B5 Lat-miR-Revertant, pRB1B5 Lat-miR- deletion, si-paip2 RNA or siRNA control as indicated. The cells were lysed 72 hours post-transfection and the extracts were analysed by immunoblotting with the indicated antibodies against viral as well as host proteins.

To further investigate the biological relevance of the viral miRNA-mediated paip2 repression during viral infection and its effect on pp14 expression, we used reverse genetics mutagenesis [35] and deleted both copies of cluster 3 miRNAs from the latency-associated region of the pRB1B5 BAC clone [36]. Cluster 3 contains miR-M6, miR-M7, miR-M8, miR-M10 and miR-M13 [35], all the 4 miRNAs that seem to mediate paip2 translation repression. Reconstruction of the mutant viruses in primary CEF transfected with the BAC DNA and analysis of *in vitro* growth kinetics show that the mutant viruses replicate with comparable kinetics, but slightly slower than the parent pRB1B5 (S6 Figure); therefore only the mutant viruses are used for this studies. Primary CEF were transfected with BAC DNA from the mutant viruses and the cells were lysed at the indicated times points with TRIzol then RNA and proteins were simultaneously extracted and analysed by immunoblotting and quantitative RT-PCR (Fig. 6). Immunoblotting shows that there is significantly higher level of pp14b isoform compared to that of pp14a isoform in CEF transfected with pRB1B5-Lat-miR-Δ and with pRB1B5-Lat-miR-Rev 48 hours post transfection (Fig. 6). The two pp14 isoforms differ by the composition of their N-termini as a result of differential splicing which gives two splice isoforms the translation of which is either cap-dependent for the pp14a or 5L IRES-driven for the pp14b [22], and as depicted in Fig. 6A. The expression level of both pp14 isoforms increases over time in CEF transfected with pRB1B5-Lat-miR-Rev (Fig. 6B); however and in contrast to pp14a, the level of pp14b decreases over time in CEF transfected with pRB1B5-Lat-miR-Δ (Fig. 6C). As judged from the quantitative real-time RT-PCR results (Fig. 6D), the differences in expression level between both pp14 isoforms cannot be solely explained by the differential accumulation of their respective transcripts that follows the same trend. We can clearly see that the levels of pp14b transcripts are consistently higher than those of the pp14a transcripts which also continue to increase over time, confirming our previous findings [22]. Interestingly, the continued increase of pp14b isoform in CEF transfected with pRB1B5-Lat-miR-Rev correlates with significant decrease in the level of paip2 protein (Fig. 6B) that itself is concomitant with increased level of MDV1 miRNAs, M7, M8, M10 and M13 (S7 Figure). There is no detectable difference over the time points examined in the abundance of paip2 mRNAs between CEF transfected with both mutant BAC DNAs (Fig. 6D). Additionally, we show that BAC DNA mutagenesis does not affect the expression of other viral miRNAs such as miR-M4 from cluster 1 (S8 Figure). These results indicate a direct link between MDV1-miRNAs expressed from the Lat-cluster and paip2-mediated translation repression. At this stage we do not know the relative contribution of each of the viral miRNAs to the overall paip2 translation repression. Additional evidence supporting the link between viral miRNAs-mediated paip2 repression and the enhanced activity of 5L IRES came from siRNA paip2-mediated repression experiments that allowed rescuing pp14b (under 5L IRES control) expression in CEF infected by pRB1B5 Lat-miR-Δ to levels comparable to those observed with pRB1B5 Lat-miR-Rev (Fig. 7). These RNAi rescue experiments clearly show that the level of paip2 is reduced by siRNA against paip2 as compared to control siRNA in CEF-infected with mutant viruses and in CEF-control, whereas the level of PABP1 expression remains unchanged under all conditions (Fig. 7). Significantly, none of these changes appear to affect the expression level of another IE protein, pp38 isoforms or the expression level of PABP1 from the host. Overall, viral miRNA-mediated paip2 repression illustrates a finely tuned transcript-specific translation control strategy that appears to specifically affect the accumulation of pp14b isoform which is under 5L IRES control, whereas the cap-dependent pp14a isoform expression remains unaffected. Our results also show that although the optimal 5L IRES activity requires the presence of PABP1, MDV1 infection does not appear to cause increased accumulation of PABP1, but the virus is instead using a strategy that ensure the availability of an active pool of PABP1.

## Discussion

**Figure 8:**
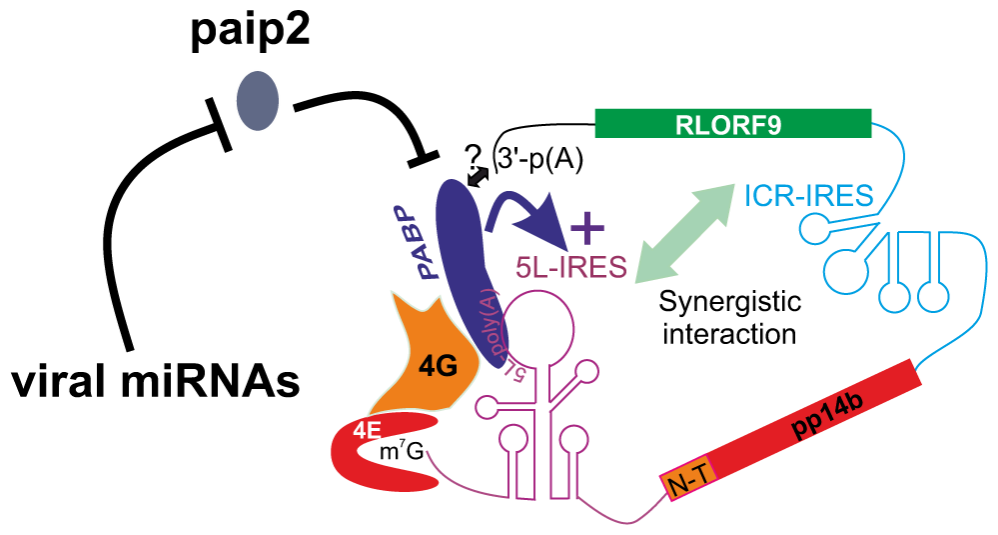
Model depicting the closed loop topology for the bicistronic immediate-early transcript (IE) that encodes RLORF9 and the pp14 from MDV1. In this model, only the pp14b isoform is shown, which is under the translation control of the 5L IRES. The internal poly(A) of the 5L IRES recruits PABP1 to the 5' end of the mRNA, which may be concomitant with the recruitment of PABP1 to the poly(A) tail of the message, leading to circularization of the bicistronic dual IRES IE-mRNA. A subset of viral miRNAs down-regulate the expression level of paip2 which is a well-known inhibitor of PABP1. This down regulation of paip2 indirectly contributes to an increased level of the available pool of active PABP1 which interacts with the internal poly(A) sequence of the 5L IRES hence leading to increased IRES activity.

As a nuclear DNA virus, MDV1 produces capped and polyadenylated mRNAs that are indistinguishable from host mRNAs. Therefore, MDV1 has to compete with the host for resources required for mRNA translation. An elegant and detailed account of how viruses gain control of key cellular signalling pathways and subvert the host protein synthesis machinery was previously reviewed [1], [2]. The temporal gene expression of MDV1 suggests that IE and late gene expression might use different strategies of translation control so that viral protein synthesis ensues with minimal disruption to the host. This is critical for MDV1 because it is a cell-associated virus and must maintain cap-dependent translation despite the inherent cellular stress caused by viral infection; and at the same time translate a subset of transcripts that require cap-independent translation initiation. When we first reported that the dominant isoform variant of the pp14-encoding bicistronic IE mRNA from MDV1 harbours the 5L IRES [22] we speculated on the possible strategies used by the virus to avoid the negative effect of the cap structure on the activity of the 5L IRES that controls the translation of pp14b isoform. Here, we provide evidence to support just such a strategy; whereby poly(A) tail-independent recruitment of the initiation factor PABP1 to an internal poly(A) within the 5L IRES specifically enhances translation. We propose a model that may offer mechanistic explanation as to how the 5L IRES activity is maintained and enhanced despite the competitive effect of the cap structure at the 5' end. In this model (Fig. 8), the internal poly(A) of the 5L IRES recruits PABP1 to the 5' end of the mRNA, which together with the well-known interaction between PABP1 and the poly(A) tail of the message, would lead to the circularization of the naturally occurring bicistronic dual IRES IE-mRNA. The circularization of the message may or may not necessarily contribute to the activity of the 5L IRES because even in the absence of the poly(A) tail the activity of the 5L IRES is maintained (Fig. 3C). Recent findings using cryo-electron tomography and showing that circular polyribosomes can be formed on eukaryotic mRNA without cap-structure and poly(A) tail [37], reinforce the validity of the “closed-loop” topology in the case of the naturally occurring bicistronic dual IRES IE-mRNA from MDV1, even though the mechanisms of non-covalent closure of the polyribosome rings still remain unsolved [37]. The recruitment of PABP1 to the 5L IRES via the internal poly(A) sequence may indirectly be facilitated by the action of virally encoded miRNAs that decrease the level of paip2, the inhibitor of PABP1, therefore leading to an increase in the available pool of active PABP1 (Fig. 8). Although not depicted in our model, the circularization may also be facilitated by protein-protein interactions between separate PABP1 molecules bound in the 5L IRES and the poly(A) tail; such interactions can be mediated by the proline- and glutamine-rich linker located between RRMs and the PABC domain as previously reported [38]. Additional work is needed to dissect the role of other major translation initiation factors such as eIF4G and how they may affect or not the activity of the 5L IRES. Nonetheless, this proposed model seems to be further supported by binding affinities data between the 5L IRES and the PABP1 as discussed below.

There are four non-identical RNA-recognition motifs (RRMs) on the PABP1, among which RRM1-2 are enough to form a continuous RNA-binding trough that accommodates 8 nucleotides from oligo(A) RNA [24]. The selectivity of RNA binding by PABP1 is partitioned between RRM1-2 and RRM3-4 domain pairs, the latter being less selective than the former [39]. The binding affinity of PABP1 with the poly(A) tail was estimated to be around 7 nM [40] which is 10-fold higher than the one we have measured for the interaction with the 5L IRES internal poly(A). The cytoplasmic concentration of PABP1 was estimated in Hella cells to be three order of magnitude above its Kd for oligo(rA)25, indicating that the PABP1 may bind to additional, lower affinity sites [40]. Our results suggest that the internal poly(A) of the 5L IRES identified in this study and in the context of infected chicken cells may well be one of these low affinity sites. This does not exclude the possibility that the internal poly(A) of the 5L IRES may also interact with RRM1-2 of PABP1. Our study reveals an alternative strategy that may facilitate the formation of mRNA “closed loop” topology without the need for the cap structure as recently shown by cryo-electron tomography [37]. Even though PABP1 is not known to be core component of the translation initiation machinery, several findings define it as a *bona fide* translation initiation factor that enhances canonical cap-dependent translation initiation by at least two mechanisms: stabilizing the molecular bridge cap-eIF4E-eIF4G-PABP-poly(A) that brings about the circularization of mRNA and stimulating 60S ribosome subunit joining [4], [41], [42]. Furthermore, unstructured poly(A) sequence within the 5' UTR was shown to mediate cap-independent, eIF4G-dependent initiation via recruitment of the PABP1 in the case of mRNAs whose translation is required for physiological adaptation to stress in yeast [43], indicating that the position of the poly(A) is less critical for the PABP1 activity, so long as it is still accessible for the interaction. This appears to be the case for the reported enhancement of viral translation by the recruitment of PABP1 to A-rich sequences embedded in structured non-adenylated 3' end of dengue virus [44]. Additionally, pre-AUG 5'-poly(A) sequence in an IRES-independent context was shown to act as translation enhancer [45] that mediates ribosomal initiation complex formation in the absence of essential initiation factors such as eIF3 and eIF4F, however, the enhancing effect of PABP1 was not investigated in those study [46]. Our data show that even in the context of a typically structured viral IRES with compact modules [22], PABP1 can still be recruited to internal poly(A) within the 5L IRES supporting the notion that extensive 5' UTR secondary structures could down regulate eIF4F binding to enable PABP1-dependent translation [47].

To ensure that an active pool of PABP1 is available for a subset of IE-mRNAs, MDV1 deploys virally-encoded miRNAs to control the level of paip2, the inhibitor of PABP1. We show that during MDV1 infection, the decrease in the level of paip2 correlates with the expression of viral miRNAs and that the level of PABP1 remains unchanged throughout the time course studied. It has been reported that under normal conditions, PABP1 homeostasis is mediated by the stability of paip2 by a mechanism that involves ubiquitin ligase, which targets paip2 for degradation [25]. Our siPABP knockdown confirms this finding. Many viruses are known for hijacking the ubiquitin system for their own benefits [48], [49]. The siPABP knockdown data reveal what might appear as a perplexing relationship between the levels of PABP1 and paip2, on the one hand, and the activity of the 5L IRES in the reporter construct on the other hand, in that the concomitant decrease in the level of paip2 (Fig. 3B) should in theory allow for sufficient free PABP1 to be available to maintain optimal 5L activity. The fact, however, that the activity of the 5L reporter is affected despite the concomitant decrease of paip2 is most likely due to the magnitude of PABP1 depletion by the siRNA which affect not only the 5L activity but also the cap-dependent translation of the reporter in which the 5L IRES is removed as seen in the reporter pR/ICR-F (S1A Figure). It is important to remember that in the context of MDV1 infection we see a decrease in the level of paip2 that appears to be mediated by viral miRNAs, see for example Fig. 4, Fig. 6 and Fig. 7. This decrease in paip2 level does not appear to have any measurable effect on the level of PABP1. It is tempting to speculate that the down-regulation of paip2 by MDV1 miRNAs might decouple the feedback loop between PABP1 and paip2 levels. This may offer a translational control advantage for the virus that enables the PABP1-mediated stimulation of 5L IRES activity thus increasing the expression of pp14b isoform without altering pp14a isoform level, which is controlled via cap-dependent translation [22]. The two isoforms differ only in their N-termini, but they have similar cellular location, and the neurovirulence phenotype is mainly associated with the C-terminus that is encoded by an exon common to both isoforms [20]. The evolution of such an elaborate translation control strategy that ensures the production of different amounts of both pp14 isoforms suggests that both proteins may offer evolutionary advantage to the virus. Accordingly, we cannot rule out the possibility that the two pp14 isoforms mediate their neurovirulence phenotype through stoichiometric interaction that is modulated by their translation levels.

## Materials and Methods

### Cell cultures, DNA constructs and transient transfection, *in vitro* transcription, PABP purification and EMSA

Primary chicken embryo fibroblast (CEF) cultures were prepared from 10-day old specific-pathogen-free embryos obtained from flock maintained at The Pirbright Institute, Compton, United Kingdom as previously described [36]. The MDV-transformed lymphoblastoid cell line MSB1 [33] and the DF-1 cell line, derived from line zero CEF [50] were cultured as described before [23]. The bicistronic dual IRES DNA construct was previously described [22] and the mutations within the 5L IRES were made synthetically (Eurogentec) and subsequently cloned into the pR/ICR-F vector upstream of the R-Luc as Pac1/Nhe1. To generate transcripts with defined poly(A_70_) tail we cloned the corresponding DNA fragment (gift from V. Mauro Laboratory, TSRI, Ca. USA) into the p5L-R/ICR-F vector as Xba1/BamH1 insert, downstream of the F-Luc. *In vitro* transcriptions of mRNAs in the presence of either 7mGpppG or ApppG were described previously [22]. Short RNA transcripts were generated using the MEGAshortscript T7 kit (Ambion) using T7-containing promoter oligonucleotides. Transient transfections with DNA and RNA luciferase reporters were carried out as before [23]. The construct for human PABP1 [51] was a gift from S. Bradrick, (Duke University, NC, USA). Untagged human PABP1 was purified using the IMPACT-CN system (New England Biolabs). ^32^P-End-labelling of the 5L IRES transcript was carried using T4 polynucleotide kinase (NEB) and gel-purified on 8% denaturing polyacrylamide gel. The labelled RNA in water was heated to 95°C for 2 min, cooled to room temperature and refolded in 10 mM Hepes-KOH, pH 7.5, 100 mM NaCl, 25 mM KCl, 2 mM MgCl2, 5% glycerol (v/v). Twenty thousand cpm (Cerenkov) of the refolded RNA was incubated for 30 min at room temperature with purified PABP1 (0.5 µM) that was diluted in the refolding buffer and supplemented with 80 µg/ml of tRNA and 10 mM DTT. For electrophoretic mobility shift assay (EMSA), samples were separated on 6% native polyacrylamide gel at 5°C followed by exposure to phosphor screen and scanning using Typhoon 9400 (GE Healthcare). For binding affinity calculation, increasing concentrations of purified recombinant human PABP1 (in 2-fold increment from 0.1–6.4 µM) were incubated with ^32^P-end labelled RNAs as described above and the bound RNA was separated from unbound using Strataclean (Stratagene) resin [52]. Briefly, 25 µl of resin slurry was washed twice with 50 µl RNA refolding buffer supplemented with RNase inhibitor (SUPERase-In, Ambion) at 1U/µl, then 20 000 cpm of refolded RNA previously mixed with increasing concentrations of PABP1 was added to the resin and incubated for ∼4 minutes. The mixture was then centrifuged at 2000×g for 1 minute and the pellet washed twice with 100 µl refolding buffer to remove unspecific binding. The amount of radioactivity corresponding to PABP1-bound to ^32^P-5L IRES in the pellet was measured for each protein concentration and data were fitted using nonlinear curve fitting to a hyperbolic equation by Graph Pad prism software.

### RNA interference, Western blotting, immunohistochemistry and antibodies

The PABP1 siRNA (sc-36169), Paip2 siRNA (sc-365317), and the control siRNA (sc-37007) were purchased from Santa Cruz. The DF-1 cells were co-transfected with the indicated bicistronic vector and siRNAs using Lipofectamine 2000 (Invitrogen) following the manufacturer protocol. Total proteins were harvested and separated using MES-NuPage Novex Bis-Tris 4–12% gel (Invitrogen) followed by transfer to PVDF membrane with the iBLOT system (Invitrogen).The antibodies user are: anti-PABP1 [10E10] (1∶1000, ab6125; Abcam), anti-paip2 (1∶500, ab33455; Abcam), anti-eIF4E (1∶500, C46H6; Cell Signaling), anti-eIF4A (1∶1000, C32B4; Cell Signaling), anti-tubulin (1∶1000, T6199; Sigma), anti-pp14 [20], anti-pp38 [20]. Detection was performed using HRP-conjugated or AP-conjugated secondary antibodies and the signals were detected with ECL-Prime or ECF, respectively (GE Healthcare). For ECF-detection of fluorescence we used using Typhoon 9400 scanner (GE Healthcare) and the signal quantified with Image Quant. Immunofluorescence staining and laser scanning microscopy were performed on CEF transfected with viral BAC DNA as described before [20]. Additional antibodies used in immunohistochemistry are mouse monoclonal anti- eIF4G [A10] (1∶400, sc-133155; Santa Cruz), rabbit polyclonal anti-eIF4G (1∶400, C45A4; Cell Signaling) and Alexa Fluor 488/568-conjugated antibodies goat anti-mouse or anti-rabbit (Invitrogen). Confocal images were taken using Leica TCS SP5 microscope. A series of optical sections were acquired sequentially for each channel along the z-axis with a step size of 0.290 µm. The images were processed and analysed with Imaris 7.5 software (Bitplane) using three-dimensional visualization.

Although no animal experiments were directly used for this work, the archive samples used for Western blotting analysis were derived from previously published work [23] that was performed in accordance with the United Kingdom Home Office guidelines under the provisions of the Project Licence approved by The Pirbright Institute Ethical Committee.

### Real time quantitative PCR and Northern blotting

Methods for quantitative RT-PCR to measure miRNA and mRNA levels have been described [22], [53]. Additional TaqMan miRNA and gene expression assays used in this study were purchased from Applied Biosystems and are: mdv1-miR-M13 (assay ID 007739_mat), mdv1-miR-M10 (assay ID 007565_mat) and paip2 (assay ID Gg03370296_m1). The primers for the 18S chicken rRNA used as endogenous control for RT-qPCR are: forward, 5'-AGAAACGGCTACCACATCCAA-3'; reverse, 5'-GGGTCGGGAGTGGGTAATTT-3' (Sigma) and the probe is: 5'-AAGGCAGCAGGCGC-3' (Applied Biosystems). Northern blotting for the detection of reporter mRNA was carried out as described before [23].

### BAC-DNA mutagenesis

Infectious BAC clone pRB-1B5 was used for the generation of the mutant constructs as previously descripted [35], [54]. The positive and negative selection marker *gal*K cassette was used in consecutive steps for the deletion of the two copies of the miRNA clusters. For this, the fragment containing the LAT miRNA cluster (GenBank EF523390 - Nucleotides 142870 to144343) was amplified with 5′-TTGTTCTGTGTTTCCTTCTC-3′ and 5′-TGATCTCCGGACCGAGAACAC-3′ primers and cloned into pGEMT vector (Promega). From this vector, the 572-bp *MscI*-*BglII* fragment EF523390 - Nucleotides 143269–143842) encompassing all the Lat miRNAs in the cluster was replaced with *gal*K cassette to generate the recombination construct for replacing the first copy of the LAT microRNAs with *gal*K. Then the *gal*K cassette was removed by replacing with the above pGEMT clone with *MscI*-*BglII* cut, Klenow filled and self-ligated plasmid. For second copy deletion, the homologous sequence from EF523390 - Nucleotides 143276-143326 with primer 5'-CACGCTATTATCCCTGCATGATCTTCTTTAATTGGACGACATTCCTCGAT CCTGTTGACAATTAATCATCGGCA-3' and the homologous sequence from EF523390 - Nucleotides 143793-143843) with primer 5'-GGACCTCTACGAGACAACGCCATCCACTAGGAAGCTTCTACGATTAAGCATCAGCACTGTCCTGCTCCTT-3' were used to PCR amplify a *gal*K cassette. As the homologous sequences in the above primers were no longer present in the deleted copy, this amplified *gal*K cassette only goes to the second copy. When the second copy was replaced with *gal*K cassette, the *gal*K cassette was further replaced with synthetic gene (GeneART) with all the pre-LAT miRNA’s loops deleted. The revertant was made on replacing the second copy of galK, with PCR product from WT sequence. Transfection of CEF with BAC DNA and reconstitution of infectious viruses was carried as described before [35].

## Supporting Information

S1 Figure **PABP1 knockdown and functional analysis of the interplay between 5L IRES internal poly(A) and poly(A) tail.** (A) DF-1 cells were co-transfected with the depicted DNA construct and with the PABP1 siRNA or nonsilencing siRNA control. The cells were lysed after 48 h incubation and used for luciferase assays. The results are presented as per cent change relative to nonsilencing siRNA control. The experiment was performed three times and the error bars indicate the SEM. Northern blotting was performed on total RNA extracted from transfected cells. Hybridization was done with a random-primed 32P-labelled DNA fragment corresponding to the 5’ end of the F-Luc open reading frame. (B) DF-1 cells were transfected for 1 h with the indicated bicistronic dual IRES mRNA reporters and subsequently washed (0 hour); then 6 hourspost transfection the luciferase activity was measured and expressed as per cent change relative to capped and polyadenylated 5Lwt-R/ICR-F mRNA. The experiment was performed three times and the error bars indicate the SEM. (D) Total RNA was extracted from the transfected cells and the integrity of the bicistronic dual IRES mRNA reporters was analysed by Northern blotting and 32 14 P-lablled probe against R-Luc, followed by phosphor screen autoradiography.
(TIF)

S2 Figure **Effect of MDV1 infection on paip2 expression.** Chicken embryo fibroblasts (CEF) were transfected with oncogenic BAC clone pRB1B5 of MDV1 or mock-transfected for 72 h. Total proteins were harvested and analysed by immunoblotting with the indicated antibodies. (A) and (B) are the results from two independent experiments.
(TIF)

S3 Figure **MDV1-infection does not appear to affect the accumulation or localization of translation initiation factors.** Indirect immunofluorescence of CEF transfected with pRB1B5 for 72 hours. Host and viral proteins were detected with indicated antibodies. A series of optical sections were taken sequentially for each channel along the z-axis using a step size of 0.290 µm. The resulting 3D confocal images were reconstructed using IMARIS software.
(TIF)

S4 Figure **List of synthetic oligonucleotides cloned as MREs in 1 psiCHECK2 vector.** Lower cases indicate the overhang for Xho1/Not1 used for cloning. The predicted MREs are shown in bold and the mutated nucleotides are underlined. **MRE1-WT**: **5’-tcga**AAGAGTAATCTGAATCCAAACGCAA**AGGAGTTTGTTCCTGGGGTGAA**GTACTTAAATATT-**3’** 3’ TTCTCATTAGACTTAGGTTTGCGTTTCCTCAAACAAGGACCCCACTTCATGAATTTATAA**ccgg**-5’ **MRE1-MT**: **5’-tcga**AAGAGTAATCTGAATCCAAACGCAA**AGGAGTTTGTTCCTGGGGattA**GTACTTAAATATT**-3’** 3’ TTCTCATTAGACTTAGGTTTGCGTTTCCTCAAACAAGGACCCCtaaTCATGAATTTATAA**ccgg**-5’ **MRE2-WT:** **5’-tcga**GGAAACATAATTGGGCCCTGG**CTCTCTGCAAAGGAGACAGTGA**GGTAGGAAGCACCAGTC-3’ 3’- CCTTTGTATTAACCCGGGACCGAGAGACGTTTCCTCTGTCACTCCATCCTTCGTGGTCAG**ccgg**-5’ **MRE2-MT:** **5’-tcga**GGAAACATAATTGGGCCCTGG**CTCTCTGCAAAGGAGACAtgcA**GGTAGGAAGCACCAGTC-3’ 3’- CCTTTGTATTAACCCGGGACCGAGAGACGTTTCCTCTGTacgTCCATCCTTCGTGGTCAG**ccgg**-5’ **MRE3-WT:** **5’-tcga**TGAGCTGTAACAGAAGTCG**TACAGACCTGCACAGAGTCATA**GATCTCAGCTACTGAACTA-3’ 3’- ACTCGACATTGTCTTCAGCATGTCTGGACGTGTCTCAGTATCTAGAGTCGATGACTTGAT**ccgg**-3’ **MRE3-MT:** **5’-tcga**TGAGCTGTAACAGAAGTCG**TACAGACCTGCACAGAGTCtat**GATCTCAGCTACTGAACTA-3’ 3’- ACTCGACATTGTCTTCAGCATGTCTGGACGTGTCTCAGataCTAGAGTCGATGACTTGAT**ccgg**-3’ **MRE4-WT:** **5’-tcga**GGAAGGGGTTCCCTGTAC**TTGCAGTATGTTATCATGTT**AGCAATGTTTCACTCCCTAATT-3’ 5’- CCTTCCCCAAGGGACATGAACGTCATACAATAGTACAATCGTTACAAAGTGAGGGATTAA**ccgg**-4’ **MRE4-MT:** **5’-tcga**GGAAGGGGTTCCCTGTAC**TTGCAGTATGTTATCAacgT**AGCAATGTTTCACTCCCTAATT-3’ 5’- CCTTCCCCAAGGGACATGAACGTCATACAATAGTtgcATCGTTACAAAGTGAGGGATTAA**ccgg**-4’
(TIF)

S5 Figure **Relative expression level of viral miRNAs in MSB1 cells as measured by TaqMan RT-PCR described in Material and methods.** The 18S rRNA is used as the endogenous control and the level miR-M4 is set to 100%.
(TIF)

S6 Figure **Growth curves of the parent pRB-1B5 BAC, the pRB-1B5 Lat-miR revertant BAC and the pRB-1B5 Lat-miR deletion BAC.** Fresh chicken embryo fibroblast were (CEF) were infected with the indicated viruses. After 0, 24, 48, 72, 96 and 120 hours the infected cultures were trypsinized and plated on fresh CEF in triplicates. MDV1 plaques were counted after visualization by immunohistochemistry.
(TIF)

S7 Figure **Relative expression of viral miRNAs within the Lat cluster from pRB1B5 Lat- miR-Revertant and pRB1B5 Lat-miR-deletion.** Chicken embryo fibroblasts were transfected with the mutant viruses as indicated and the RNA was extracted using Trizol at the indicated time points and subsequently used for quantitative RT-PCR to check the expression of MDV1-miRNAs. The 18S rRNA is the endogenous control and time zero is the calibrator. All experiments were repeated three times and the error bars indicate the SEM.
(TIF)

S8 Figure **Reverse genetic manipulation does not alter the expression of MDV1 miRNA outside Lat-cluster.** Chicken embryo fibroblasts (CEF) were transfected with BAC clone pRB1B5 Lat-miR- Revertant or pRB1B5 Lat-miR- deletion. The RNA was extracted using Trizol at the indicated time points and used for quantitative RT-PCR to check the expression of MDV1-miR-M4 at the indicated time points. The 18S rRNA is the endogenous control and time zero is the calibrator. All experiments were repeated three times and the error bars indicate the SEM.
(TIF)

## References

[1] Buchkovich NJ, Yu Y, Zampieri CA, Alwine JC (2008) The TORrid affairs of viruses: effects of mammalian DNA viruses on the PI3K-Akt-mTOR signalling pathway. Nat Rev Microbiol 6:266–275. 18311165 10.1038/nrmicro1855 PMC2597498

[2] Walsh D, Mohr I (2011) Viral subversion of the host protein synthesis machinery. Nat Rev Microbiol 9:860–875. 22002165 10.1038/nrmicro2655 PMC7097311

[3] Burgess HM, Gray NK (2010) mRNA-specific regulation of translation by poly(A)-binding proteins. Biochem Soc Trans 38:1517–1522. 21118118 10.1042/BST0381517

[4] Wells SE, Hillner PE, Vale RD, Sachs AB (1998) Circularization of mRNA by eukaryotic translation initiation factors. Mol Cell 2:135–140. 9702200 10.1016/s1097-2765(00)80122-7

[5] Jackson RJ, Hellen CU, Pestova TV (2010) The mechanism of eukaryotic translation initiation and principles of its regulation. Nat Rev Mol Cell Biol 11:113–127. 20094052 10.1038/nrm2838 PMC4461372

[6] Smith RW, Gray NK (2010) Poly(A)-binding protein (PABP): a common viral target. Biochem J 426:1–12. 20102337 10.1042/BJ20091571

[7] Joachims M, Van Breugel PC, Lloyd RE (1999) Cleavage of poly(A)-binding protein by enterovirus proteases concurrent with inhibition of translation in vitro. J Virol 73:718–727. 9847378 10.1128/jvi.73.1.718-727.1999 PMC103879

[8] Kerekatte V, Keiper BD, Badorff C, Cai A, Knowlton KU, et al (1999) Cleavage of Poly(A)-binding protein by coxsackievirus 2A protease in vitro and in vivo: another mechanism for host protein synthesis shutoff? J Virol 73:709–717. 9847377 10.1128/jvi.73.1.709-717.1999 PMC103878

[9] Harb M, Becker MM, Vitour D, Baron CH, Vende P, et al (2008) Nuclear localization of cytoplasmic poly(A)-binding protein upon rotavirus infection involves the interaction of NSP3 with eIF4G and RoXaN. J Virol 82:11283–11293. 18799579 10.1128/JVI.00872-08 PMC2573281

[10] Piron M, Vende P, Cohen J, Poncet D (1998) Rotavirus RNA-binding protein NSP3 interacts with eIF4GI and evicts the poly(A) binding protein from eIF4F. EMBO J 17:5811–5821. 9755181 10.1093/emboj/17.19.5811 PMC1170909

[11] Blakqori G, van Knippenberg I, Elliott RM (2009) Bunyamwera orthobunyavirus S-segment untranslated regions mediate poly(A) tail-independent translation. J Virol 83:3637–3646. 19193790 10.1128/JVI.02201-08 PMC2663239

[12] Arias C, Walsh D, Harbell J, Wilson AC, Mohr I (2009) Activation of host translational control pathways by a viral developmental switch. PLoS Pathog 5:e1000334. 19300492 10.1371/journal.ppat.1000334 PMC2652079

[13] Dobrikova E, Shveygert M, Walters R, Gromeier M (2010) Herpes simplex virus proteins ICP27 and UL47 associate with polyadenylate-binding protein and control its subcellular distribution. J Virol 84:270–279. 19864386 10.1128/JVI.01740-09 PMC2798443

[14] Salaun C, MacDonald AI, Larralde O, Howard L, Lochtie K, et al (2010) Poly(A)-binding protein 1 partially relocalizes to the nucleus during herpes simplex virus type 1 infection in an ICP27-independent manner and does not inhibit virus replication. J Virol 84:8539–8548. 20573819 10.1128/JVI.00668-10 PMC2919032

[15] Burgess HM, Richardson WA, Anderson RC, Salaun C, Graham SV, et al (2011) Nuclear relocalisation of cytoplasmic poly(A)-binding proteins PABP1 and PABP4 in response to UV irradiation reveals mRNA-dependent export of metazoan PABPs. J Cell Sci 124:3344–3355. 21940797 10.1242/jcs.087692 PMC3178455

[16] Kumar GR, Shum L, Glaunsinger BA (2011) Importin alpha-mediated nuclear import of cytoplasmic poly(A) binding protein occurs as a direct consequence of cytoplasmic mRNA depletion. Mol Cell Biol 31:3113–3125. 21646427 10.1128/MCB.05402-11 PMC3147611

[17] Perez C, McKinney C, Chulunbaatar U, Mohr I (2011) Translational control of the abundance of cytoplasmic poly(A) binding protein in human cytomegalovirus-infected cells. J Virol 85:156–164. 20980505 10.1128/JVI.01778-10 PMC3014207

[18] Walsh D, Perez C, Notary J, Mohr I (2005) Regulation of the translation initiation factor eIF4F by multiple mechanisms in human cytomegalovirus-infected cells. J Virol 79:8057–8064. 15956551 10.1128/JVI.79.13.8057-8064.2005 PMC1143722

[19] McKinney C, Perez C, Mohr I (2012) Poly(A) binding protein abundance regulates eukaryotic translation initiation factor 4F assembly in human cytomegalovirus-infected cells. Proc Natl Acad Sci U S A 109:5627–5632. 22431630 10.1073/pnas.1202829109 PMC3326494

[20] Tahiri-Alaoui A, Smith LP, Kgosana L, Petherbridge LJ, Nair V (2013) Identification of a neurovirulence factor from Marek's disease virus. Avian Diseases 57:387–394. 23901751 10.1637/10322-080912-Reg.1

[21] Osterrieder N, Kamil JP, Schumacher D, Tischer BK, Trapp S (2006) Marek's disease virus: from miasma to model. Nat Rev Microbiol 4:283–294. 16541136 10.1038/nrmicro1382

[22] Tahiri-Alaoui A, Matsuda D, Xu H, Panagiotis P, Burman L, et al (2009) The 5' leader of the mRNA encoding the marek's disease virus serotype 1 pp14 protein contains an intronic internal ribosome entry site with allosteric properties. J Virol 83:12769–12778. 19793814 10.1128/JVI.01010-09 PMC2786844

[23] Tahiri-Alaoui A, Smith LP, Baigent S, Kgosana L, Petherbridge LJ, et al (2009) Identification of an intercistronic internal ribosome entry site in a Marek's disease virus immediate-early gene. J Virol 83:5846–5853. 19297480 10.1128/JVI.02602-08 PMC2681985

[24] Deo RC, Bonanno JB, Sonenberg N, Burley SK (1999) Recognition of polyadenylate RNA by the poly(A)-binding protein. Cell 98:835–845. 10499800 10.1016/s0092-8674(00)81517-2

[25] Yoshida M, Yoshida K, Kozlov G, Lim NS, De Crescenzo G, et al (2006) Poly(A) binding protein (PABP) homeostasis is mediated by the stability of its inhibitor, Paip2. EMBO J 25:1934–1944. 16601676 10.1038/sj.emboj.7601079 PMC1456944

[26] Thoma C, Bergamini G, Galy B, Hundsdoerfer P, Hentze MW (2004) Enhancement of IRES-mediated translation of the c-myc and BiP mRNAs by the poly(A) tail is independent of intact eIF4G and PABP. Mol Cell 15:925–935. 15383282 10.1016/j.molcel.2004.08.021

[27] Thoma C, Fraterman S, Gentzel M, Wilm M, Hentze MW (2008) Translation initiation by the c-myc mRNA internal ribosome entry sequence and the poly(A) tail. RNA 14:1579–1589. 18556416 10.1261/rna.1043908 PMC2491467

[28] Khaleghpour K, Svitkin YV, Craig AW, DeMaria CT, Deo RC, et al (2001) Translational repression by a novel partner of human poly(A) binding protein, Paip2. Mol Cell 7:205–216. 11172725 10.1016/s1097-2765(01)00168-x

[29] Miranda KC, Huynh T, Tay Y, Ang YS, Tam WL, et al (2006) A pattern-based method for the identification of MicroRNA binding sites and their corresponding heteroduplexes. Cell 126:1203–1217. 16990141 10.1016/j.cell.2006.07.031

[30] Morgan R, Anderson A, Bernberg E, Kamboj S, Huang E, et al (2008) Sequence conservation and differential expression of Marek's disease virus microRNAs. J Virol 82:12213–12220. 18842708 10.1128/JVI.01722-08 PMC2593341

[31] Yao Y, Zhao Y, Xu H, Smith LP, Lawrie CH, et al (2008) MicroRNA profile of Marek's disease virus-transformed T-cell line MSB-1: predominance of virus-encoded microRNAs. J Virol 82:4007–4015. 18256158 10.1128/JVI.02659-07 PMC2293013

[32] Burnside J, Bernberg E, Anderson A, Lu C, Meyers BC, et al (2006) Marek's disease virus encodes MicroRNAs that map to meq and the latency-associated transcript. J Virol 80:8778–8786. 16912324 10.1128/JVI.00831-06 PMC1563840

[33] Akiyama Y, Kato S (1974) Two cell lines from lymphomas of Marek's disease. Biken J 17:105–116. 4616680

[34] Zhao Y, Yao Y, Xu H, Lambeth L, Smith LP, et al (2009) A functional MicroRNA-155 ortholog encoded by the oncogenic Marek's disease virus. J Virol 83:489–492. 18945769 10.1128/JVI.01166-08 PMC2612317

[35] Zhao Y, Xu H, Yao Y, Smith LP, Kgosana L, et al (2011) Critical role of the virus-encoded microRNA-155 ortholog in the induction of Marek's disease lymphomas. PLoS Pathog 7:e1001305. 21383974 10.1371/journal.ppat.1001305 PMC3044692

[36] Petherbridge L, Brown AC, Baigent SJ, Howes K, Sacco MA, et al (2004) Oncogenicity of virulent Marek's disease virus cloned as bacterial artificial chromosomes. J Virol 78:13376–13380. 15542691 10.1128/JVI.78.23.13376-13380.2004 PMC525015

[37] Afonina ZA, Myasnikov AG, Shirokov VA, Klaholz BP, Spirin AS (2014) Formation of circular polyribosomes on eukaryotic mRNA without cap-structure and poly(A)-tail: a cryo electron tomography study. Nucleic Acids Res 42:9461–9469. 25016525 10.1093/nar/gku599 PMC4132722

[38] Melo EO, Dhalia R, Martins de Sa C, Standart N, de Melo Neto OP (2003) Identification of a C-terminal poly(A)-binding protein (PABP)-PABP interaction domain: role in cooperative binding to poly (A) and efficient cap distal translational repression. J Biol Chem 278:46357–46368. 12952955 10.1074/jbc.M307624200

[39] Sladic RT, Lagnado CA, Bagley CJ, Goodall GJ (2004) Human PABP binds AU-rich RNA via RNA-binding domains 3 and 4. Eur J Biochem 271:450–457. 14717712 10.1046/j.1432-1033.2003.03945.x

[40] Gorlach M, Burd CG, Dreyfuss G (1994) The mRNA poly(A)-binding protein: localization, abundance, and RNA-binding specificity. Exp Cell Res 211:400–407. 7908267 10.1006/excr.1994.1104

[41] Kahvejian A, Svitkin YV, Sukarieh R, M'Boutchou MN, Sonenberg N (2005) Mammalian poly(A)-binding protein is a eukaryotic translation initiation factor, which acts via multiple mechanisms. Genes Dev 19:104–113. 15630022 10.1101/gad.1262905 PMC540229

[42] Sonenberg N, Hinnebusch AG (2009) Regulation of translation initiation in eukaryotes: mechanisms and biological targets. Cell 136:731–745. 19239892 10.1016/j.cell.2009.01.042 PMC3610329

[43] Gilbert WV, Zhou K, Butler TK, Doudna JA (2007) Cap-independent translation is required for starvation-induced differentiation in yeast. Science 317:1224–1227. 17761883 10.1126/science.1144467

[44] Polacek C, Friebe P, Harris E (2009) Poly(A)-binding protein binds to the non-polyadenylated 3' untranslated region of dengue virus and modulates translation efficiency. J Gen Virol 90:687–692. 19218215 10.1099/vir.0.007021-0

[45] Gudkov AT, Ozerova MV, Shiryaev VM, Spirin AS (2005) 5'-poly(A) sequence as an effective leader for translation in eukaryotic cell-free systems. Biotechnol Bioeng 91:468–473. 15986488 10.1002/bit.20525

[46] Shirokikh NE, Spirin AS (2008) Poly(A) leader of eukaryotic mRNA bypasses the dependence of translation on initiation factors. Proc Natl Acad Sci U S A 105:10738–10743. 18658239 10.1073/pnas.0804940105 PMC2485544

[47] Svitkin YV, Evdokimova VM, Brasey A, Pestova TV, Fantus D, et al (2009) General RNA-binding proteins have a function in poly(A)-binding protein-dependent translation. EMBO J 28:58–68. 19078965 10.1038/emboj.2008.259 PMC2633083

[48] Isaacson MK, Ploegh HL (2009) Ubiquitination, ubiquitin-like modifiers, and deubiquitination in viral infection. Cell Host Microbe 5:559–570. 19527883 10.1016/j.chom.2009.05.012 PMC7103382

[49] Jarosinski K, Kattenhorn L, Kaufer B, Ploegh H, Osterrieder N (2007) A herpesvirus ubiquitin-specific protease is critical for efficient T cell lymphoma formation. Proc Natl Acad Sci U S A 104:20025–20030. 18056809 10.1073/pnas.0706295104 PMC2148416

[50] Himly M, Foster DN, Bottoli I, Iacovoni JS, Vogt PK (1998) The DF-1 chicken fibroblast cell line: transformation induced by diverse oncogenes and cell death resulting from infection by avian leukosis viruses. Virology 248:295–304. 9721238 10.1006/viro.1998.9290

[51] Bradrick SS, Dobrikova EY, Kaiser C, Shveygert M, Gromeier M (2007) Poly(A)-binding protein is differentially required for translation mediated by viral internal ribosome entry sites. RNA 13:1582–1593. 17652408 10.1261/rna.556107 PMC1950770

[52] Sayer NM, Cubin M, Rhie A, Bullock M, Tahiri-Alaoui A, et al (2004) Structural determinants of conformationally selective, prion-binding aptamers. J Biol Chem 279:13102–13109. 14711834 10.1074/jbc.M310928200

[53] Xu H, Yao Y, Zhao Y, Smith LP, Baigent SJ, et al (2008) Analysis of the expression profiles of Marek's disease virus-encoded microRNAs by real-time quantitative PCR. J Virol Methods 149:201–208. 18355930 10.1016/j.jviromet.2008.02.005

[54] Zhao Y, Nair V (2010) Mutagenesis of the repeat regions of herpesviruses cloned as bacterial artificial chromosomes. Methods Mol Biol 634:53–74. 20676975 10.1007/978-1-60761-652-8_4

