# Supplementary figures and images for: Poly(A) Binding Protein 1 Enhances Cap-Independent Translation Initiation of Neurovirulence Factor from Avian Herpesvirus
Source: PLoS One. 2014 Dec 11;9(12):e114466. doi: 10.1371/journal.pone.0114466 (PMC4263670; doi:10.1371/journal.pone.0114466)

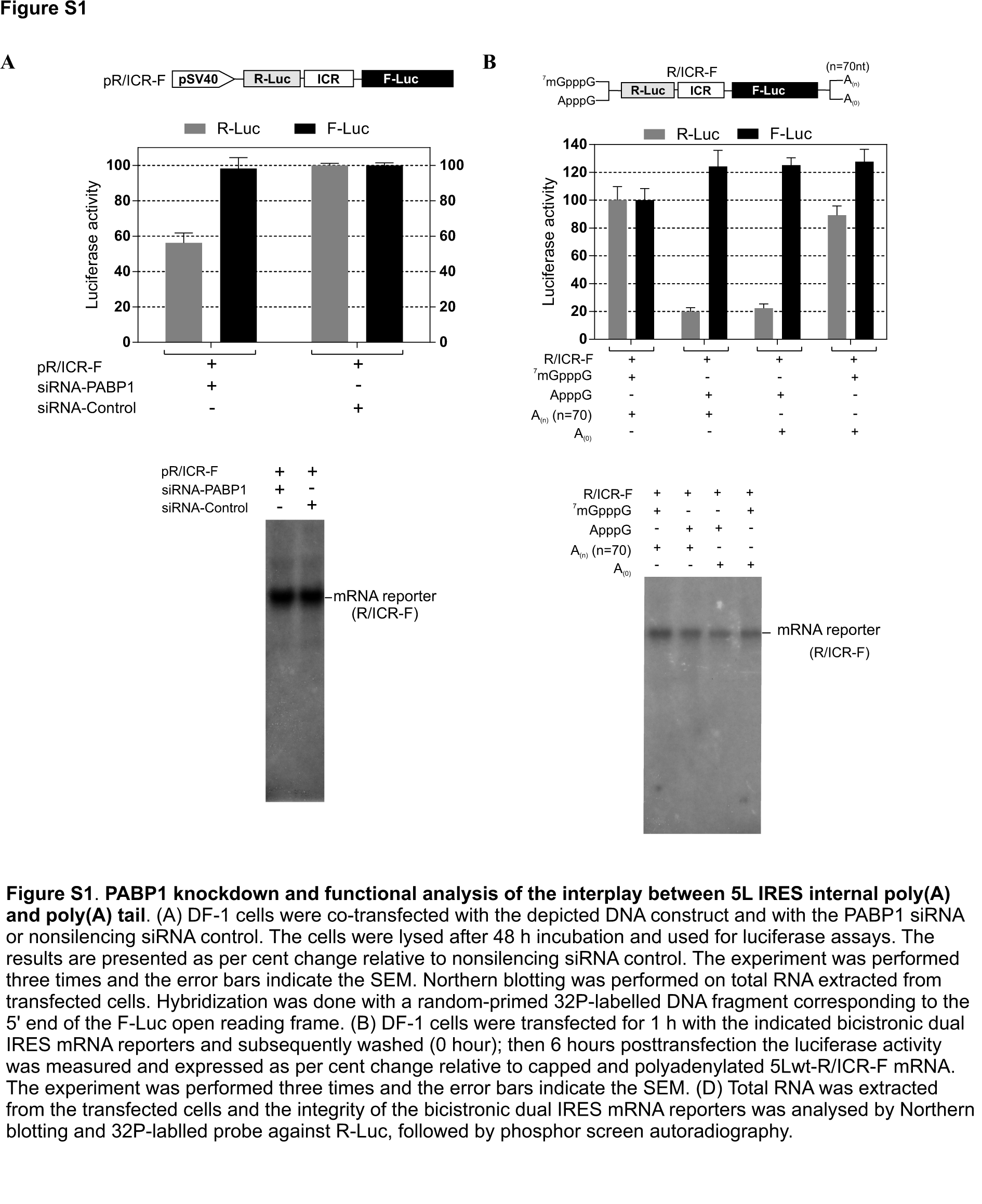

Supplement: S1 Figure — PABP1 knockdown and functional analysis of the interplay between 5L IRES internal poly(A) and poly(A) tail. (A) DF-1 cells were co-transfected with the depicted DNA construct and with the PABP1 siRNA or nonsilencing siRNA control. The cells were lysed after 48 h incubation and used for luciferase assays. The results are presented as per cent change relative to nonsilencing siRNA control. The experiment was performed three times and the error bars indicate the SEM. Northern blotting was performed on total RNA extracted from transfected cells. Hybridization was done with a random-primed 32P-labelled DNA fragment corresponding to the 5’ end of the F-Luc open reading frame. (B) DF-1 cells were transfected for 1 h with the indicated bicistronic dual IRES mRNA reporters and subsequently washed (0 hour); then 6 hourspost transfection the luciferase activity was measured and expressed as per cent change relative to capped and polyadenylated 5Lwt-R/ICR-F mRNA. The experiment was performed three times and the error bars indicate the SEM. (D) Total RNA was extracted from the transfected cells and the integrity of the bicistronic dual IRES mRNA reporters was analysed by Northern blotting and 32 14 P-lablled probe against R-Luc, followed by phosphor screen autoradiography. (TIF) [file pone.0114466.s001.tif]

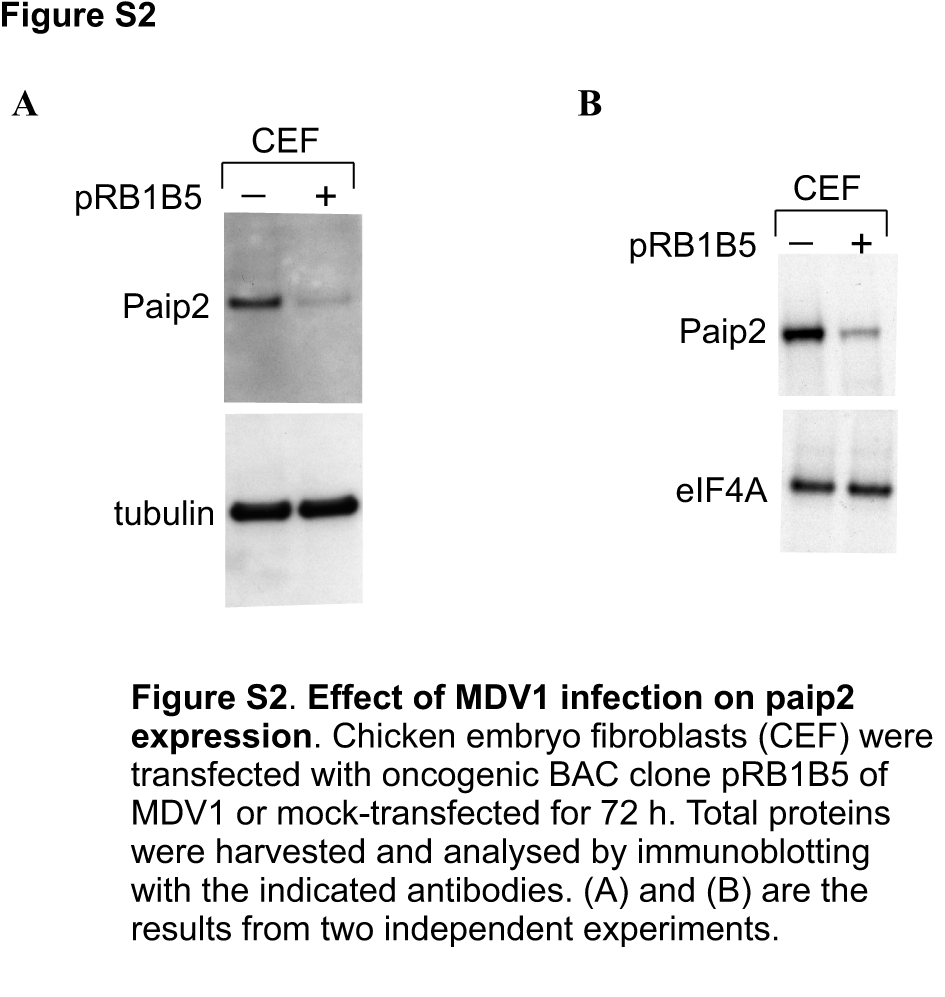

Supplement: S2 Figure — Effect of MDV1 infection on paip2 expression. Chicken embryo fibroblasts (CEF) were transfected with oncogenic BAC clone pRB1B5 of MDV1 or mock-transfected for 72 h. Total proteins were harvested and analysed by immunoblotting with the indicated antibodies. (A) and (B) are the results from two independent experiments. (TIF) [file pone.0114466.s002.tif]

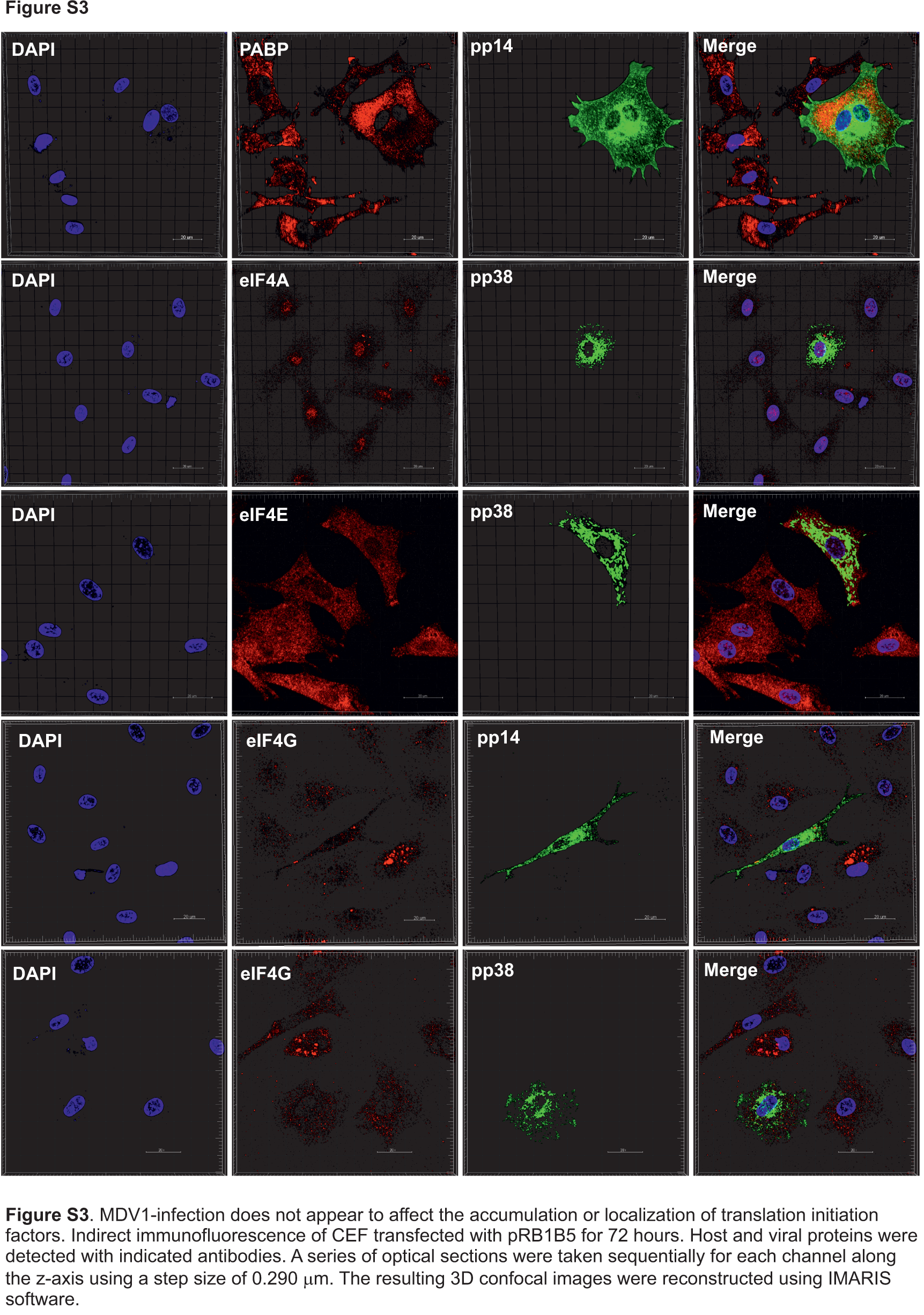

Supplement: S3 Figure — MDV1-infection does not appear to affect the accumulation or localization of translation initiation factors. Indirect immunofluorescence of CEF transfected with pRB1B5 for 72 hours. Host and viral proteins were detected with indicated antibodies. A series of optical sections were taken sequentially for each channel along the z-axis using a step size of 0.290 µm. The resulting 3D confocal images were reconstructed using IMARIS software. (TIF) [file pone.0114466.s003.tif]

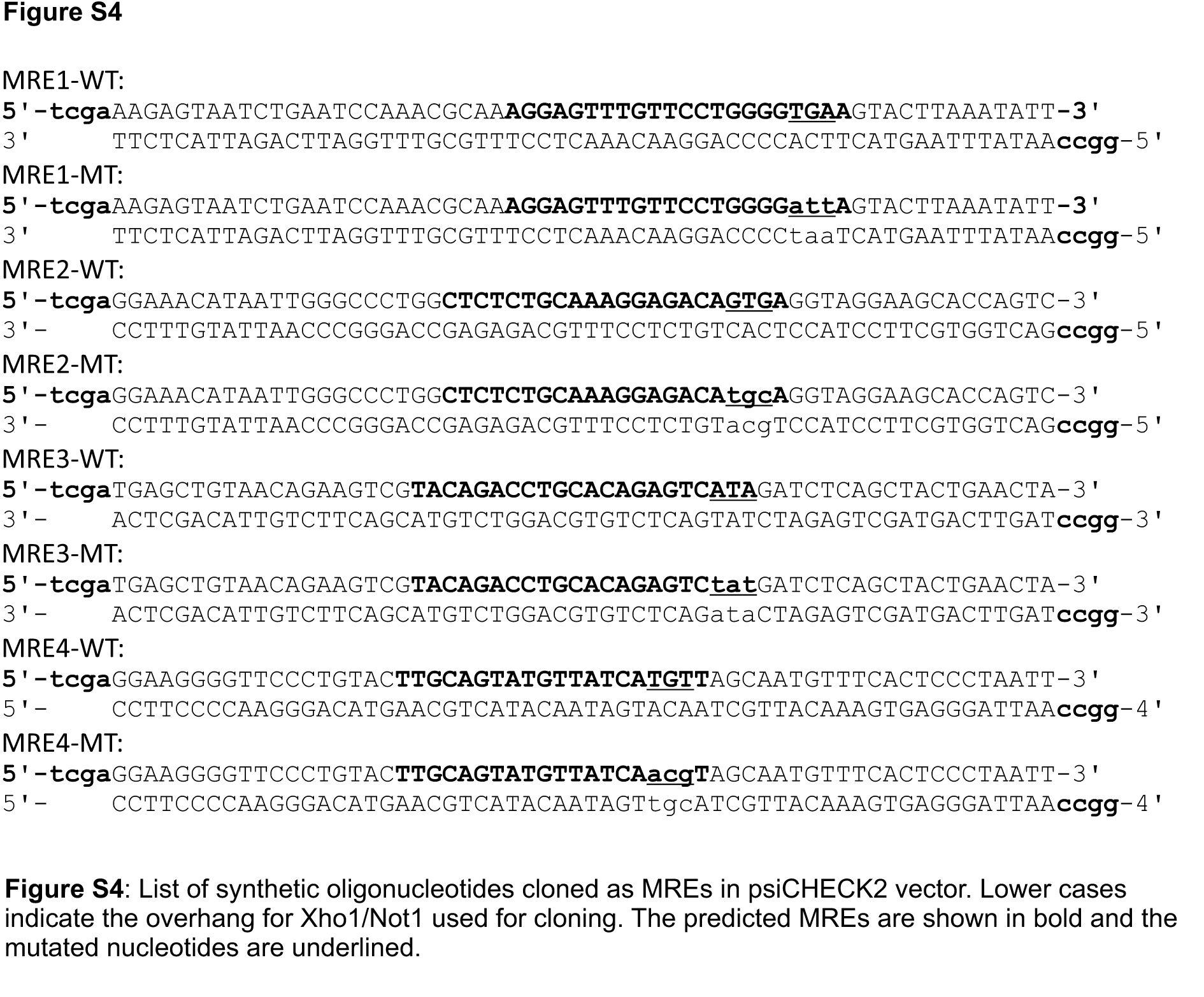

Supplement: S4 Figure — List of synthetic oligonucleotides cloned as MREs in 1 psiCHECK2 vector. Lower cases indicate the overhang for Xho1/Not1 used for cloning. The predicted MREs are shown in bold and the mutated nucleotides are underlined. MRE1-WT: 5’-tcgaAAGAGTAATCTGAATCCAAACGCAAAGGAGTTTGTTCCTGGGGTGAAGTACTTAAATATT-3’ 3’ TTCTCATTAGACTTAGGTTTGCGTTTCCTCAAACAAGGACCCCACTTCATGAATTTATAAccgg-5’ MRE1-MT: 5’-tcgaAAGAGTAATCTGAATCCAAACGCAAAGGAGTTTGTTCCTGGGGattAGTACTTAAATATT-3’ 3’ TTCTCATTAGACTTAGGTTTGCGTTTCCTCAAACAAGGACCCCtaaTCATGAATTTATAAccgg-5’ MRE2-WT: 5’-tcgaGGAAACATAATTGGGCCCTGGCTCTCTGCAAAGGAGACAGTGAGGTAGGAAGCACCAGTC-3’ 3’- CCTTTGTATTAACCCGGGACCGAGAGACGTTTCCTCTGTCACTCCATCCTTCGTGGTCAGccgg-5’ MRE2-MT: 5’-tcgaGGAAACATAATTGGGCCCTGGCTCTCTGCAAAGGAGACAtgcAGGTAGGAAGCACCAGTC-3’ 3’- CCTTTGTATTAACCCGGGACCGAGAGACGTTTCCTCTGTacgTCCATCCTTCGTGGTCAGccgg-5’ MRE3-WT: 5’-tcgaTGAGCTGTAACAGAAGTCGTACAGACCTGCACAGAGTCATAGATCTCAGCTACTGAACTA-3’ 3’- ACTCGACATTGTCTTCAGCATGTCTGGACGTGTCTCAGTATCTAGAGTCGATGACTTGATccgg-3’ MRE3-MT: 5’-tcgaTGAGCTGTAACAGAAGTCGTACAGACCTGCACAGAGTCtatGATCTCAGCTACTGAACTA-3’ 3’- ACTCGACATTGTCTTCAGCATGTCTGGACGTGTCTCAGataCTAGAGTCGATGACTTGATccgg-3’ MRE4-WT: 5’-tcgaGGAAGGGGTTCCCTGTACTTGCAGTATGTTATCATGTTAGCAATGTTTCACTCCCTAATT-3’ 5’- CCTTCCCCAAGGGACATGAACGTCATACAATAGTACAATCGTTACAAAGTGAGGGATTAAccgg-4’ MRE4-MT: 5’-tcgaGGAAGGGGTTCCCTGTACTTGCAGTATGTTATCAacgTAGCAATGTTTCACTCCCTAATT-3’ 5’- CCTTCCCCAAGGGACATGAACGTCATACAATAGTtgcATCGTTACAAAGTGAGGGATTAAccgg-4’ (TIF) [file pone.0114466.s004.tif]

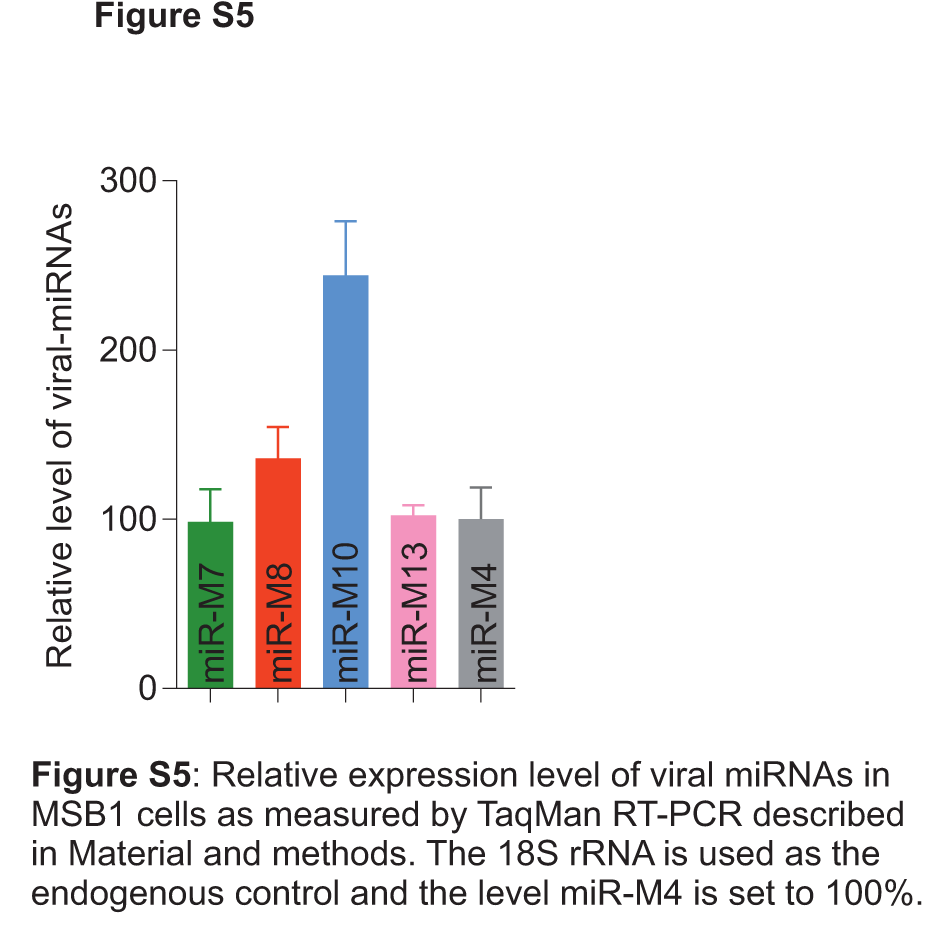

Supplement: S5 Figure — Relative expression level of viral miRNAs in MSB1 cells as measured by TaqMan RT-PCR described in Material and methods. The 18S rRNA is used as the endogenous control and the level miR-M4 is set to 100%. (TIF) [file pone.0114466.s005.tif]

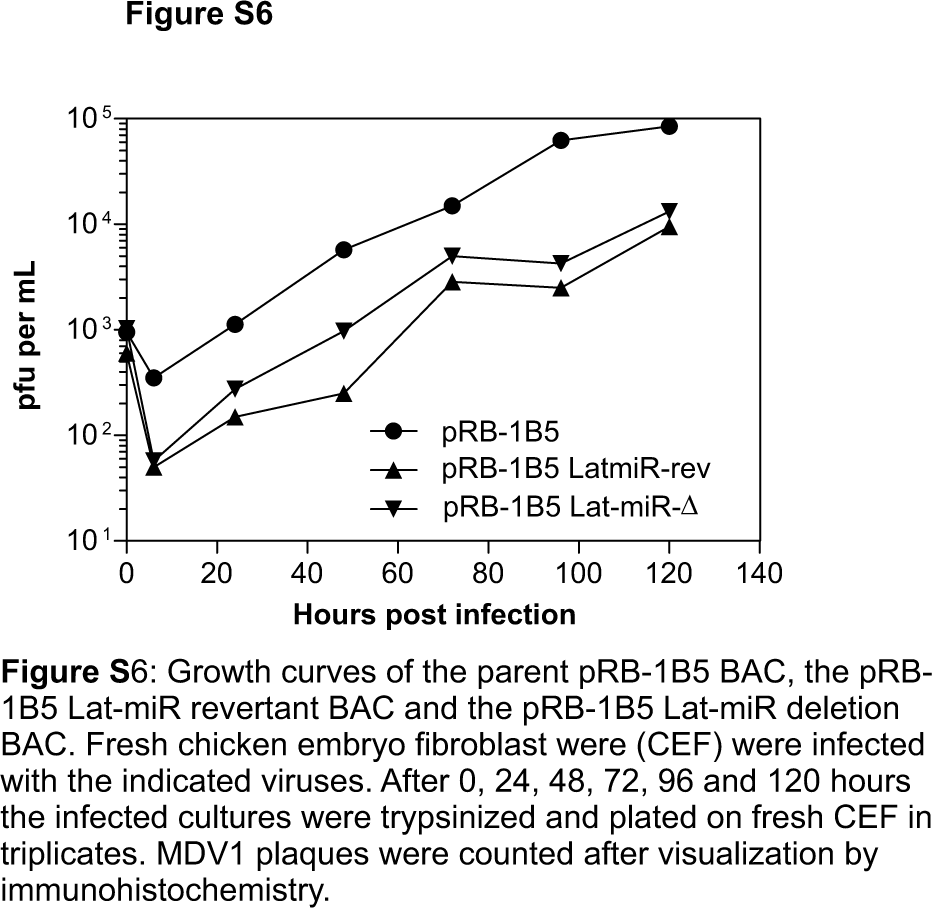

Supplement: S6 Figure — Growth curves of the parent pRB-1B5 BAC, the pRB-1B5 Lat-miR revertant BAC and the pRB-1B5 Lat-miR deletion BAC. Fresh chicken embryo fibroblast were (CEF) were infected with the indicated viruses. After 0, 24, 48, 72, 96 and 120 hours the infected cultures were trypsinized and plated on fresh CEF in triplicates. MDV1 plaques were counted after visualization by immunohistochemistry. (TIF) [file pone.0114466.s006.tif]

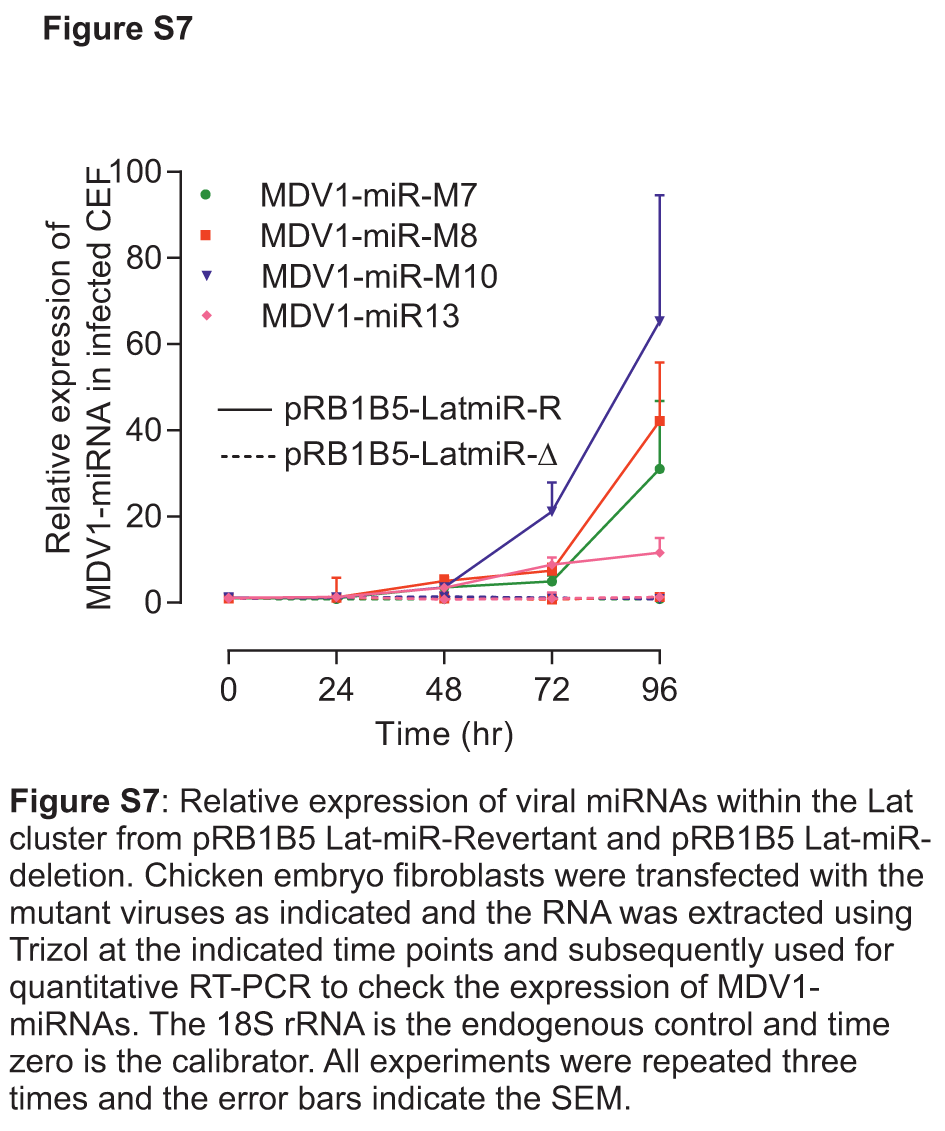

Supplement: S7 Figure — Relative expression of viral miRNAs within the Lat cluster from pRB1B5 Lat- miR-Revertant and pRB1B5 Lat-miR-deletion. Chicken embryo fibroblasts were transfected with the mutant viruses as indicated and the RNA was extracted using Trizol at the indicated time points and subsequently used for quantitative RT-PCR to check the expression of MDV1-miRNAs. The 18S rRNA is the endogenous control and time zero is the calibrator. All experiments were repeated three times and the error bars indicate the SEM. (TIF) [file pone.0114466.s007.tif]

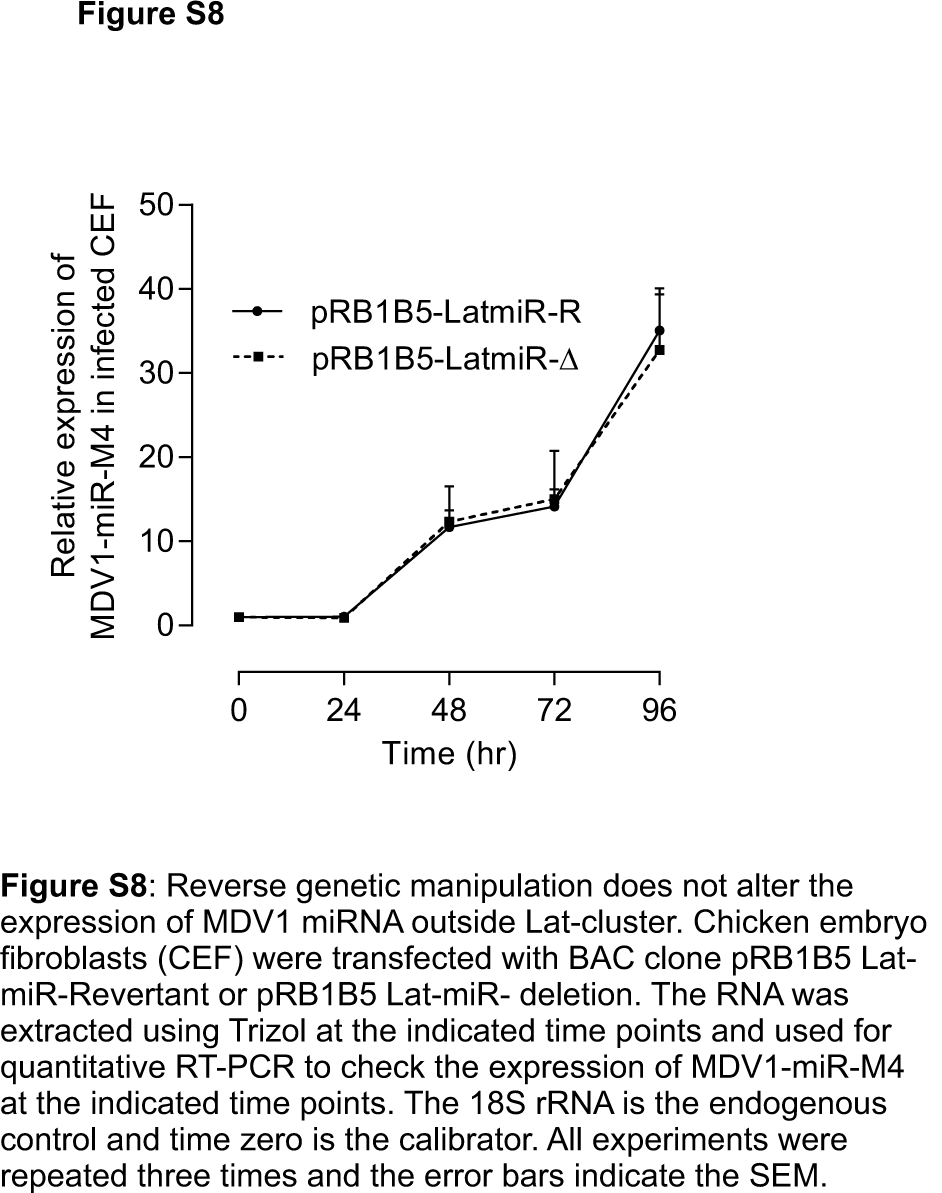

Supplement: S8 Figure — Reverse genetic manipulation does not alter the expression of MDV1 miRNA outside Lat-cluster. Chicken embryo fibroblasts (CEF) were transfected with BAC clone pRB1B5 Lat-miR- Revertant or pRB1B5 Lat-miR- deletion. The RNA was extracted using Trizol at the indicated time points and used for quantitative RT-PCR to check the expression of MDV1-miR-M4 at the indicated time points. The 18S rRNA is the endogenous control and time zero is the calibrator. All experiments were repeated three times and the error bars indicate the SEM. (TIF) [file pone.0114466.s008.tif]
